# Correction: Behavioral and genetic correlates of heterogeneity in learning performance in individual honeybees, *Apis mellifera*

**DOI:** 10.1371/journal.pone.0312715

**Published:** 2024-10-23

**Authors:** The PLOS ONE Staff

## Notice of republication

This article was republished on October 9, 2024, to correct an error in the author list: Gérard Leboulle was erroneously displayed as Leboulle. The publisher apologizes for this error. Please download this article again to view the correct version. The originally published, uncorrected article and the republished, corrected article are provided here for reference

## Supporting information

S1 File Originally published, uncorrected article. (PDF)

S2 File Republished, corrected article. (PDF)
