# Supplementary material for: Behavioral and genetic correlates of heterogeneity in learning performance in individual honeybees, Apis mellifera
Source: PLoS One. 2024 Jun 12;19(6):e0304563. doi: 10.1371/journal.pone.0304563 (PMC11168654; doi:10.1371/journal.pone.0304563)
Supplement: S1 Fig — RT-qPCR analysis of expression differences of the 9-selected gene candidates (represented by respective standard IDs): Comparison of the mRNA levels of the selected genes between the high and low performer bees. Each bar represents the relative mRNA levels +/- SE in the low (green) and high (red) performers, normalized to the levels of the low performers. The differences in expression levels measured by microarray are also given (yellow bars). (DOCX) [file pone.0304563.s001.docx]

**S1 Fig. RT-qPCR analysis of the nine-candidate genes for the high and low performer bees.**


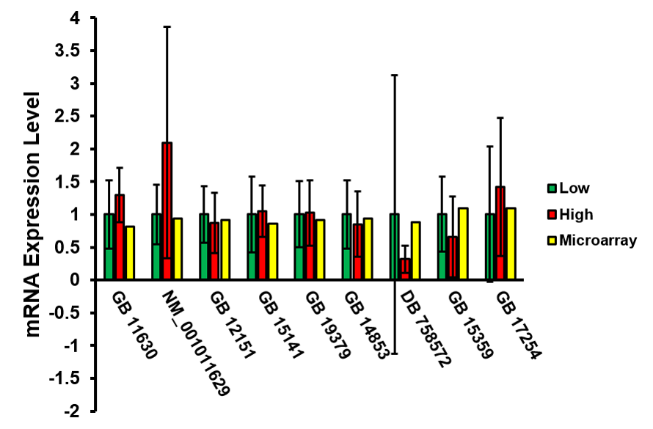


RT-qPCR analysis of expression differences of the 9-selected gene candidates (represented by respective standard IDs): Comparison of the mRNA levels of the selected genes between the high and low performer bees. Each bar represents the relative mRNA levels +/- SE in the low (green) and high (red) performers, normalized to the levels of the low performers. The differences in expression levels measured by microarray are also given (yellow bars).
