# Supplementary material for: Behavioral and genetic correlates of heterogeneity in learning performance in individual honeybees, Apis mellifera
Source: PLoS One. 2024 Jun 12;19(6):e0304563. doi: 10.1371/journal.pone.0304563 (PMC11168654; doi:10.1371/journal.pone.0304563)
Supplement: S1 Table — (DOCX) [file pone.0304563.s002.docx]

**S1 Table.** **List of primer sequences for the selected transcripts used for RT-qPCR analysis.**

| **Primer** | **5’-3’ Sequence** |
| --- | --- |
| GB12151F1 | ACGTTGTTTTGTTTGACAGTG |
| GB12151R1 | GGAGAACTGAATGGTCATCATAC |
| GB14853F2 | AAGGAAGCTGGCGAAAGGATAAC |
| GB14853R2 | CCAAGCAGGAAATAACAATGAACC |
| DB758572F1 | AAAGCCAAAAGTACATATTTTTATGAA |
| DB758572R1 | CTAAGTGGCAGTTAGTCGTTGAGAAT |
| GB17254F1 | GATCCACAGTACAGCTCGGCAGTCA |
| GB17254R1 | GGGCGACGATTGCGATTGG |
| GB17254-act-F2 | TGCCAACACTGTCCTTTCTG |
| VACHT-actR-f | AGAATTGACCCACCAATCCA |
| NM_001011629F2 | AGCAGCAGCAACAACTGAACCAT |
| NM_001011629R2 | GTTGCGGCTGCTGTTGTTGAGT |
| GB15141F2 | TCGCCGATTTCGTCAAGGTTTT |
| GB15141R2 | GTGAAGCTCTCCGGAAGGCAGTA |
| GB19379F1 | GCCATATTGCGTGCTTGTGTTTG |
| GB19379R1 | GGCTTCGGGTTTTTGTTCCTGAT |
| GB15359F1 | GCATCGCAGCGTAGGTTGAA |
| GB15359R1 | CTGTTGCCATGTGTAAGTGTAAAT |
| NM_001185146.1_F actin | TTGTATGCCAACACTGTCCTTT |
| NM_001185146.1_R actin | TGGCGCGATGATCTTAATTT |
