# Supplementary material for: Behavioral and genetic correlates of heterogeneity in learning performance in individual honeybees, Apis mellifera
Source: PLoS One. 2024 Jun 12;19(6):e0304563. doi: 10.1371/journal.pone.0304563 (PMC11168654; doi:10.1371/journal.pone.0304563)
Supplement: S2 Table — Fisher statistic values (Stimulus × Trial interaction effect) and p-values of the Tukey HSD posthoc test (after Bonferroni correction of p-value) are given for the four sets of retention test, performed during the two phases of sequential learning. All p-values, except one, represented in bold and with asterisk, are significant. Note that the p-value is nonsignificant (bold and asterisk) for the highest dilutions of the odor pair, OA and LA only during the 4th retention test. (DOCX) [file pone.0304563.s003.docx]

**S2 Table.** **Results of the repeated measures ANOVA for the four sets of retention tests of the pooled population.**

| **Retention Test** | **Stimulus × Trial Effect & posthoc Test (Tukey HSD)** |
| --- | --- |
| 1^st^ | Stimulus × Trial: F_2,680_ = 26.74, p < 0.001  Tukey HSD posthoc test:  CS+ (10^-3^) vs. CS- (10^-3^): p < 0.016  CS+ (10^-2^) vs. CS- (10^-2^): p < 0.016  CS+ (Pure) vs. CS- (Pure): p < 0.016 |
| 2^nd^ | Stimulus × Trial: F_2,680_ = 30.5, p < 0.001  Tukey HSD posthoc test:  CS+ (10^-3^) vs. CS- (10^-3^): p < 0.016  CS+ (10^-2^) vs. CS- (10^-2^): p < 0.016  CS+ (Pure) vs. CS- (Pure): p < 0.016 |
| 3^rd^ | Stimulus × Trial: F_2,680_ = 52.33, p < 0.001  Tukey HSD posthoc test:  CS+ (10^-3^) vs. CS- (10^-3^): p < 0.016  CS+ (10^-2^) vs. CS- (10^-2^): p < 0.016  CS+ (Pure) vs. CS- (Pure): p < 0.016 |
| 4^th^ | Stimulus × Trial: F_2,680_ = 80.95, p < 0.001  Tukey HSD posthoc test:  CS+ (10^-3^) vs. CS- (10^-3^): **p > 0.016**  CS+ (10^-2^) vs. CS- (10^-2^): p < 0.016  CS+ (Pure) vs. CS- (Pure): p < 0.016 |

Fisher statistic values (Stimulus × Trial interaction effect) and *p*-values of the Tukey HSD posthoc test (after Bonferroni correction of *p*-value) are given for the four sets of retention test, performed during the two phases of sequential learning. All *p*-values, except one, represented in bold, are significant. Note that the *p*-value is nonsignificant (bold and asterisk) for the highest dilutions of the odor pair, OA and LA only during the 4^th^ retention test.
