# Supplementary material for: Behavioral and genetic correlates of heterogeneity in learning performance in individual honeybees, Apis mellifera
Source: PLoS One. 2024 Jun 12;19(6):e0304563. doi: 10.1371/journal.pone.0304563 (PMC11168654; doi:10.1371/journal.pone.0304563)
Supplement: S3 Table — Fisher statistic values (Stimulus × Trial interaction effect and Stimulus main effect are shown respectively for the two sets of differential conditioning and four sets of retention tests) and p-values of the Tukey HSD posthoc test are given for the high and low performer bees. Both the high and low performer bees showed significant Stimulus × Trial and Stimulus effects however, no significant difference is found between the conditioned responses to the CS+ and CS- odors during the first differential conditioning for the low performers. Bonferroni-corrected p-values (Differential conditioning: p < 0.008, Retention test: p < 0.016) are used for comparisons in the posthoc test. Significant and non-significant p-values (posthoc test) are respectively represented with asterisks and in bold (see Fig 5). For the differential conditioning, only the trials with significant p-values (posthoc test) are given. (DOCX) [file pone.0304563.s004.docx]

**S3 Table. Results of the repeated measures ANOVA for the two phases of conditioning and four sets of retention tests for the high and low performer bees.**

| **Group** | **1^st^**  **Differential**  **Conditioning** | **2^nd^ Differential**  **Conditioning** | **1^st^ Retention Test** | **2^nd^ Retention Test** | **3^rd^ Retention Test** | **4^th^ Retention Test** |
| --- | --- | --- | --- | --- | --- | --- |
| **High Performer Bees** | Stimulus × Trial:  F_5,1170_ = 104.85,  p < 0.001  Tukey HSD  posthoc test:  2^nd^ Trial: CS+ vs. CS-  *(p < 0.008)  3^rd^ Trial: CS+ vs. CS-  *(p < 0.008)  4^th^ Trial: CS+  vs. CS-  *(p < 0.008)  5^th^ Trial: CS+ vs. CS-  *(p < 0.008)  6^th^ Trial: CS+ vs. CS-  *(p < 0.008) | Stimulus × Trial:  F_5,1170_ = 63.37,  p < 0.001  Tukey HSD  posthoc test:  4^th^ Trial: CS+ vs. CS-  *(p < 0.008)  5^th^ Trial: CS+ vs. CS-  *(p < 0.008)  6^th^ Trial: CS+ vs. CS-  *(p < 0.008) | Stimulus:  F_1,234_ = 564.76,  p < 0.001  Tukey HSD posthoc test:  CS+ (10^-3^) vs. CS- (10^-3^):  *p < 0.016  CS+ (10^-2^) vs. CS- (10^-2^):  *p < 0.016  CS+ (Pure) vs. CS- (Pure):  *p < 0.016 | Stimulus:  F_1,234_ = 341.15,  p < 0.001  Tukey HSD posthoc test:  CS+ (10^-3^) vs. CS- (10^-3^):  *p < 0.016  CS+ (10^-2^) vs. CS- (10^-2^):  *p < 0.016  CS+ (Pure) vs. CS- (Pure):  *p < 0.016 | Stimulus:  F_1,234_ = 206.78,  p < 0.001  Tukey HSD posthoc test:  CS+ (10^-3^) vs. CS- (10^-3^):  *p < 0.016  CS+ (10^-2^) vs. CS- (10^-2^):  *p < 0.016  CS+ (Pure) vs. CS- (Pure):  *p < 0.016 | Stimulus:  F_1,234_ = 186.17,  p < 0.001  Tukey HSD posthoc test:  CS+ (10^-3^) vs. CS- (10^-3^):  **p > 0.016**  CS+ (10^-2^) vs. CS- (10^-2^):  *p < 0.016  CS+ (Pure) vs. CS- (Pure):  *p < 0.016 |
| **Low Performer Bees** | Stimulus × Trial:  F_5,520_ = 6.81,  p < 0.001  Tukey HSD  posthoc test:  No significant difference | Stimulus × Trial:  F_5,520_ = 19.73,  p < 0.001  Tukey HSD  posthoc test:  5^th^ Trial: CS+ vs. CS- *(p < 0.008)  6^th^ Trial: CS+ vs. CS-  *(p < 0.008) | Stimulus:  F_1,104_ = 32,  p < 0.001  Tukey HSD posthoc test:  CS+ (10^-3^) vs. CS- (10^-3^):  *p < 0.016  CS+ (10^-2^) vs. CS- (10^-2^):  *p < 0.016  CS+ (Pure) vs. CS- (Pure):  *p < 0.016 | Stimulus:  F_1,104_ = 28.27,  p < 0.001  Tukey HSD posthoc test:  CS+ (10^-3^) vs. CS- (10^-3^):  **p > 0.016**  CS+ (10^-2^) vs. CS- (10^-2^):  **p > 0.016**  CS+ (Pure) vs. CS- (Pure):  *p < 0.016 | Stimulus:  F_1,104_ = 28.93,  p < 0.001  Tukey HSD posthoc test:  CS+ (10^-3^) vs. CS- (10^-3^):  **p > 0.016**  CS+ (10^-2^) vs. CS- (10^-2^):  *p < 0.016  CS+ (Pure) vs. CS- (Pure):  *p < 0.016 | Stimulus:  F_1,104_ = 22.82,  p < 0.001  Tukey HSD posthoc test:  CS+ (10^-3^) vs. CS- (10^-3^):  **p > 0.016**  CS+ (10^-2^) vs. CS- (10^-2^):  **p > 0.016**  CS+ (Pure) vs. CS- (Pure):  *p < 0.016 |

Fisher statistic values (Stimulus × Trial interaction effect and Stimulus main effect are shown respectively for the two sets of differential conditioning and four sets of retention tests) and *p*-values of the Tukey HSD posthoc test are given for the high and low performer bees. Both the high and low performer bees showed significant Stimulus × Trial and Stimulus effects however, no significant difference is found between the conditioned responses to the CS+ and CS- odors during the first differential conditioning for the low performers. Bonferroni-corrected *p*-values (Differential conditioning: *p* < 0.008, Retention test: *p* < 0.016) are used for comparisons in the posthoc test. Significant and non-significant *p*-values (posthoc test) are respectively represented with asterisks and in bold (see Figure 5). For the differential conditioning, only the trials with significant *p*-values (posthoc test) are given.
