# Supplementary material for: Behavioral and genetic correlates of heterogeneity in learning performance in individual honeybees, Apis mellifera
Source: PLoS One. 2024 Jun 12;19(6):e0304563. doi: 10.1371/journal.pone.0304563 (PMC11168654; doi:10.1371/journal.pone.0304563)
Supplement: S4 Table — Correlation table for the seven variables quantified from the pooled data of bees. High correlation coefficient values (in bold) are found between the following pairs: Acq-1 and DisCond-1 (ρ = 0.88), Acq-1 and P-score (ρ = 0.85), DisCond-1 and P-score (ρ = 0.85), DisT-1,-2 and P-score (ρ = 0.7) and Acq-2 and P-score (ρ = 0.69). All correlations are significant (**correlations are significant at the 0.01 level, *correlations are significant at the 0.05 level). (DOCX) [file pone.0304563.s005.docx]

**S4 Table. Spearman rank order correlation coefficients between the seven variables.**

|  | **Acq-1** | **Acq-2** | **DisCond-1** | **DisCond-2** | **DisT-1,-2** | **DisT-3,-4** | **P-score** |
| --- | --- | --- | --- | --- | --- | --- | --- |
| **Acq-1** |  | 0.53** | **0.88**** | 0.14* | 0.58** | 0.34** | **0.85**** |
| **Acq-2** | 0.53** |  | 0.44** | 0.42** | 0.35** | 0.30** | **0.69**** |
| **DisCond-1** | 0.88** | 0.44** |  | 0.23** | 0.56** | 0.34** | **0.85**** |
| **DisCond-2** | 0.14* | 0.42** | 0.23** |  | 0.15* | 0.31** | 0.48** |
| **DisT-1,-2** | 0.58** | 0.35** | 0.56** | 0.15* |  | 0.35** | **0.7**** |
| **DisT-3,-4** | 0.34** | 0.3** | 0.34** | 0.31** | 0.35** |  | 0.57** |
| **P-score** | **0.85**** | **0.69**** | **0.85**** | 0.48** | **0.7**** | 0.57** |  |

Correlation table for the seven variables quantified from the pooled data of bees. High correlation coefficient values (in bold) are found between the following pairs: Acq-1 and DisCond-1 (ρ = 0.88), Acq-1 and P-score (ρ = 0.85), DisCond-1 and P-score (ρ = 0.85), DisT-1,-2 and P-score (ρ = 0.7) and Acq-2 and P-score (ρ = 0.69). All correlations are significant (**correlations are significant at the 0.01 level, *correlations are significant at the 0.05 level).
