# Supplementary material for: Behavioral and genetic correlates of heterogeneity in learning performance in individual honeybees, Apis mellifera
Source: PLoS One. 2024 Jun 12;19(6):e0304563. doi: 10.1371/journal.pone.0304563 (PMC11168654; doi:10.1371/journal.pone.0304563)
Supplement: S5 Table — Selection of the high performer bees, from the four colonies, from the different months of the experimental year (2010) for the analysis of colony effect. The column ‘Bee’ is showing the identification number of the bees. (DOCX) [file pone.0304563.s006.docx]

**S5 Table. High performer bees for the analysis of colony effect.**

| **P-Score** | **Bee** | **Colony** | **Month** | **Dye** |
| --- | --- | --- | --- | --- |
| 3.96 | A_04 P1 | 67 | July | Cy5 |
|  |  |  |  | Cy3 |
| 4.9 | B_03 P2 | 73 | July | Cy5 |
|  |  |  |  | Cy3 |
| 4.46 | C_06 P1 | 98 | July | Cy5 |
|  |  |  |  | Cy3 |
| 4.66 | D_04 P2 | 299 | July | Cy5 |
|  |  |  |  | Cy3 |
| 4.0 | A_08 P1 | 67 | August | Cy5 |
|  |  |  |  | Cy3 |
| 4.66 | A_06 P1 | 67 | August | Cy5 |
|  |  |  |  | Cy3 |
| 4.93 | B_010 P2 | 73 | August | Cy5 |
|  |  |  |  | Cy3 |
| 5.06 | C_08 P1 | 98 | August | Cy5 |
|  |  |  |  | Cy3 |
| 4.46 | D_09 P1 | 299 | August | Cy5 |
|  |  |  |  | Cy3 |
| 5.3 | B_026 P1 | 73 | October | Cy5 |
|  |  |  |  | Cy3 |
| 5.3 | C_025 P2 | 98 | October | Cy5 |
|  |  |  |  | Cy3 |
| 4.7 | D_025 P2 | 299 | October | Cy5 |
|  |  |  |  | Cy3 |

Selection of the high performer bees, from the four colonies, from the different months of the experimental year (2010) for the analysis of colony effect. The column ‘Bee’ is showing the identification number of the bees.
