# Supplementary material for: Behavioral and genetic correlates of heterogeneity in learning performance in individual honeybees, Apis mellifera
Source: PLoS One. 2024 Jun 12;19(6):e0304563. doi: 10.1371/journal.pone.0304563 (PMC11168654; doi:10.1371/journal.pone.0304563)
Supplement: S9 Table — The list of selected bees (high and low performers), for the validation by reverse transcription quantitative real-time PCR, from the four colonies and from different months of the experimental year. (DOCX) [file pone.0304563.s010.docx]

**S9 Table. List of bees selected for the validation by RT-qPCR.**

| **P-Score** | **Bee** | **Colony** | **Month** |
| --- | --- | --- | --- |
| 0 | A_012 P1 | 67 | August |
| 0 | A_011 P2 | 67 | August |
| 4.6 | A_06 P2 | 67 | August |
| 4.9 | A_026 P2 | 67 | October |
| 4.16 | B_01 P2 | 73 | July |
| 2.96 | B_011 P1 | 73 | August |
| 2.76 | B_021 P1 | 73 | September |
| 3.83 | B_020 P1 | 73 | September |
| 0.33 | C_02 P2 | 98 | July |
| 3.33 | C_015 P1 | 98 | September |
| 4.53 | C_015 P2 | 98 | September |
| 5.46 | C_016 P2 | 98 | September |
| 4.56 | D_02 P1 | 299 | July |
| 2.6 | D_023 P2 | 299 | September |
| 0.76 | D_020 P1 | 299 | September |
| 4.93 | D_024 P1 | 299 | October |

The list of selected bees (high and low performers), for the validation by reverse transcription quantitative real-time PCR, from the four colonies and from different months of the experimental year.
