# Supplementary material for: Correction: Behavioral and genetic correlates of heterogeneity in learning performance in individual honeybees, Apis mellifera
Source: PLoS One. 2024 Oct 23;19(10):e0312715. doi: 10.1371/journal.pone.0312715 (PMC11498677; doi:10.1371/journal.pone.0312715)
Supplement: S2 File — (PDF) [file pone.0312715.s002.pdf]

PLOS ONE

RESEARCH ARTICLE

Behavioral and genetic correlates of heterogeneity in learning performance in individual honeybees, *Apis mellifera*

Neloy Kumar Chakroborty[1☯¤]*, Gérard Leboulle[1☯], Ralf Einspanier[2], Randolf Menzel[1]*

1 Institute Biology, Neurobiology, Freie Universität Berlin, Königin Luisestr, Berlin, Germany, **2** Department of Veterinary Medicine, Institute of Veterinary Biochemistry, Freie Universität Berlin, Oertzenweg, Berlin, Germany

☯ These authors contributed equally to this work.
¤ Current address: Thapar School of Liberal Arts & Sciences, Thapar Institute of Engineering & Technology, Prem Nagar, Patiala, Punjab, India
* neloy.chakroborty@thapar.edu (NKC); menzel@neurobiologie.fu-berlin.de (RM)

Check for updates

OPEN ACCESS

Citation: Chakroborty NK, Leboulle G, Einspanier R, Menzel R (2024) Behavioral and genetic correlates of heterogeneity in learning performance in individual honeybees, *Apis mellifera*. PLoS ONE 19(6): e0304563. https://doi.org/10.1371/journal.pone.0304563

Editor: Mathieu Lihoreau, CNRS, University Paul Sabatier, FRANCE

Received: March 2, 2024

Accepted: May 13, 2024

Published: June 12, 2024

Copyright: © 2024 Chakroborty et al. This is an open access article distributed under the terms of the Creative Commons Attribution License, which permits unrestricted use, distribution, and reproduction in any medium, provided the original author and source are credited.

Data Availability Statement: All relevant data are within the manuscript and its Supporting information files.

Funding: The author(s) received no specific funding for this work.

Competing interests: Authors have declared that no competing interest exists

Abstract

Learning an olfactory discrimination task leads to heterogeneous results in honeybees with some bees performing very well and others at low rates. Here we investigated this behavioral heterogeneity and asked whether it was associated with particular gene expression patterns in the bee's brain. Bees were individually conditioned using a sequential conditioning protocol involving several phases of olfactory learning and retention tests. A cumulative score was used to differentiate the tested bees into high and low performers. The rate of CS + odor learning was found to correlate most strongly with a cumulative performance score extracted from all learning and retention tests. Microarray analysis of gene expression in the mushroom body area of the brains of these bees identified a number of differentially expressed genes between high and low performers. These genes are associated with diverse biological functions, such as neurotransmission, memory formation, cargo trafficking and development.

Introduction

Some of the pivotal components of animal cognition are the adaptability to new environmental conditions, capacity to maintain successful changes in behavior caused by new learning, and the use of memories for further behavioral control. In honeybees, a classical model of learning and memory studies, a large body of data on how these cognitive functions are essential for their foraging success and survival of their colonies has been documented [1, 2]. Evolutionary selection for the improvement of cognitive abilities work on the individual level and are based on genetic variations that are favorable for behavioral traits related to sensory processing, learning and memory formation [3, 4]. The search for such cognitive adaptations can be performed both on a population level and on an individual level. However, our current understanding of the ecological and evolutionary drivers of intraspecific variability in cognitive

abilities and the consequences of this variability on the individuals and populations is restricted [5–9] due to limited research work done on these topics. Studies across different vertebrate species recognized consistent among-individual variability in cognitive capacities [10–14], albeit tracing invertebrate cognition through the lens of individual variability in behavior is a much less explored territory, in spite of the increasing evidence of incredible cognitive abilities of invertebrates [1, 15–17].

Selection for behavioral traits usually involve many animals. Studies on learning and memory in *Drosophila* have led to the discovery of multiple genes related to learning and memory [18]; however, the olfactory conditioning procedures usually engage many individuals at the same time, e.g. in the T-maze paradigm [19], which makes it impossible to relate individual performances to their corresponding gene expression patterns. Olfactory conditioning protocols in *Drosophila* used by others also failed to decipher the heterogeneity in the expression of learned behavior among the individuals. Instead, the results were interpreted as the expression of probabilistic behavior at the individual level [20, 21]. Honeybees, on the other hand, can be selected for fast learning and then selectively bred via the thelytokous development of *Apis mellifera capensis* [22]; a highly effective procedure that can be applied more frequently to understand the contribution of genetic variability to the variation observed in cognitive ability. Tait and colleagues [23] tested individual honeybees for associative acquisition, non-associative sensitization, and short-term memory, and constructed a general heritable factor similar to the factors derived from mammals [24–26]. The proboscis extension response (PER) training is an effective experimental procedure that allows testing individual honeybees for a whole range of associative, non-associative and rule learning related paradigms in well controlled sequence of events [27, 28]. However, learning and memory retention scores are expressed in all these studies as average scores of the groups of animals. Motivated by the study of Gallistel and coworkers [29], Pamir and colleagues [30] analyzed a large dataset on PER conditioning in the honeybee and found that once an animal started eliciting the conditioned response (CR), it retained the CR in the subsequent conditioning trials with a high probability. Importantly, the gradual acquisition in the groups of animals that results from the recruitment of individuals to the learner population, as predicted by Gallistel et al. [29], was also found for the honyebees. Additionally, individual animals switched abruptly to a learned state during the acquisition phase after several acquisition trials, and then continued performing as learners [30]. Further, another study reported that bees switching to the state of learner early during the acquisition phase performed better in the tests of memory retention, even though they did not discriminate better between the rewarded and unrewarded conditioned stimuli during the differential conditioning [31]. These results have revealed some of the important aspects of learning behaviour of individuals, which were previously unnoticed in the analyses of group-averaged learning data. However, no clear picture has emerged on the different classes of honeybees that learn odours with different efficacies.

We therefore attempted to quantify this population heterogeneity, with the goal to understand the factor(s) that structures this among-individual heterogeneity, by a sequential conditioning procedure that allows for finer classification of individuals on the behavioral level based on their performances during two subsequent phases of differential olfactory conditioning and several retention tests; evaluating the strength of olfactory learning and sensitivity. Gene expression patterns were then determined in the brains of the honeybees that belonged to different performer classes.

Multiple studies had previously linked different behaviors in honeybees to their genetic background, such as the developmentally-regulated division of labor in the colony [32–34], learning and memory processes in adult bees [35–37], egg-laying and foraging behavior of the workers in queenless colonies [38], specialized tasks such as guarding and undertaking [39],

foraging [40, 41], scouting and recruiting [42] as well as hygienic behavior against the *Varroa* mite [43]. It has also been shown that manipulation of transcript levels in the honeybee brain influences memory performances [44–46]. Furthermore, a comparative transcriptomic study in the wasp, *Polistes metricus* also revealed genetic associations of behavioral tasks and substantial overlap of genes involved in the division of labor in the honeybee and foraging in the wasp [47]. In the harvester ant, *Pogonomyrmex californicus*, large differences in gene expression, including genes involved in neuronal functions and chemosensory perception, also have been reported for those queens showing different degrees of aggressive behavior [48]. We therefore selected the honeybees based on our previous work on their multiple forms of learning and memory functions and the brain parts involved in these performances [1, 49], particularly the mushroom bodies for genetic analysis, which are known to be involved in higher order processing of information, learning and memory in both the bee and *Drosophila* [50–52]. The aim of our study was to investigate the nature of behavioral heterogeneity that prevails in the natural populations of honeybees and to access whether the heterogeneity is associated with the expression patterns of genes in the brains of honeybees.

Materials and methods

Honeybee lines

A backcrossed genetic line of honeybees (*Apis mellifera carnica*), selected for improved hygienic behavior, was used in this study (Länderinstitut für Bienenkunde (LIB), Hohen Neuendorf, Berlin [53]. This line was generated by artificially inseminating a queen from a non-hygienic (+/+) colony with the sperms from the hygienic drones (H). Heterozygous (H/+) queens were selectively raised from the F1 progeny and backcrossed with the sperms of their hygienic (H) paternal populations. These backcrossed heterozygous (H/+) queens then were used to raise four colonies, (colony 67, 73, 98 and 299) characterized by worker bees with homozygous (H/H) and heterozygous (H/+) genetic backgrounds for the hygienic trait. It is expected that these backcrossed colonies have more homogenous genetic backgrounds than the natural colonies, which is advantageous for the behavioral and genetic investigations. Indeed, previous studies have revealed that heterogeneity in olfactory learning performance of honeybees has a genetic basis [54–56].

The experiment took place between July and October 2010. During the course of our experiment, colony 67 became aggressive and was quarantined. From the time point of its confinement, we excluded a total of 32 bees from this colony from the data analysis.

Procedure for sequential olfactory PER conditioning

Forager honeybees were caught at the entrance of the colonies on the day before sequential conditioning. Bees were harnessed in small plastic tubes as described elsewhere [27, 57, 58], fed until satiation with 30% (W/V) sucrose solution and kept overnight inside a humid Styrofoam box in the dark. The next morning, they were placed in front of the experimental arena for 30–45 min for adaptation. The sequential conditioning protocol consisted of two identical phases, each composed of one round of differential conditioning followed by two sets of retention tests (Fig 1). During the differential conditioning, bees were trained to learn and discriminate an odor (conditioned stimulus, CS+) paired forwardly with the presentation of a sucrose reward (a 30% sucrose solution was used as unconditioned stimulus, US) from a non-reinforced odor (CS–) in the course of 12 consecutive conditioning trials (6 CS+ and 6 CS–). The two pure odor stimuli were presented alternately starting with the CS+ with an inter-trial interval (time gap between consecutive CS+ and CS–trials) of 8 min. During the CS+ trials, the odor was presented for 5 s and the US was applied 3 s after the odor onset by first touching the

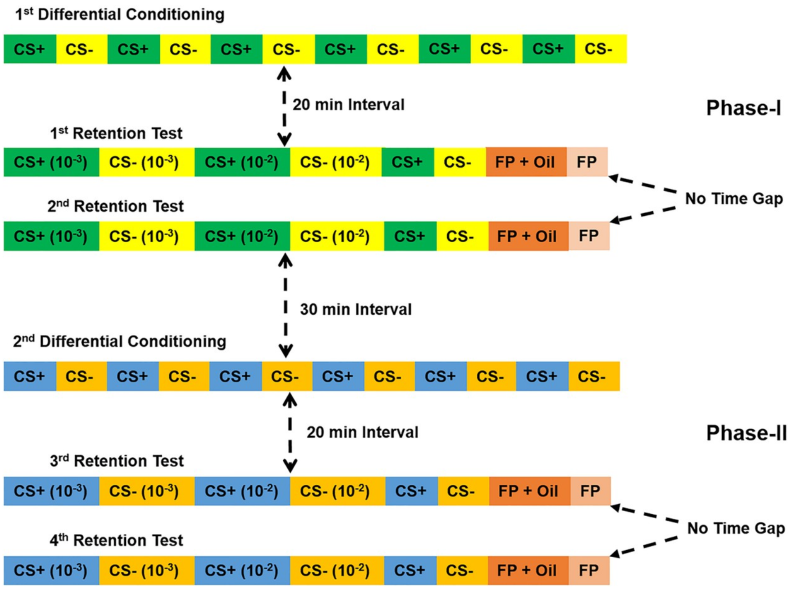

Fig 1. The protocol of sequential conditioning.

https://doi.org/10.1371/journal.pone.0304563.g001

antenna with a toothpick soaked with the sucrose solution to elicit proboscis extension, followed by feeding through the proboscis for a total of 4 s. Thus, there was an overlap of 2 s between the odor stimulation and the US. During the CS–trials and the retention tests, the odors were presented alone for 5 s. During conditioning, a PER elicited during the first 3 s of odor stimulation before the onset of the US was considered as a conditioned response (CR). A 20 min break was given after the differential conditioning, which followed two sets of retention tests. In each test, bees were presented with increasing concentrations of the CS+ and CS– odors on filter paper (10^{-3} and 10^{-2} dilutions in paraffin oil and the pure odors) starting with the CS+. Each set of retention test was terminated by stimulating the animals once with paraffin oil and once with the filter paper alone. There was no time gap between the two consecutive sets of retention tests. The second round of differential conditioning began after a pause of 30 min and a different odor pair was used (Fig 1). The ability of the bee to elicit a CR was evaluated by visualizing a PER after stimulation of the antennae with sugar water at the end of the fourth set of retention tests. Only bees that elicited PER were considered for data analysis. Bees were sacrificed immediately after this fitness-test, by placing them at –20˚C for 10 min followed by preservation at –80˚C until the gene expression study. The whole procedure took a total time of 6 h and 30 min to complete.

The protocol includes two phases, each composed of one differential olfactory conditioning and two sets of retention tests. Conditioning was performed with the pure odors (CS+, CS-) and the retention tests with increasing odor concentrations, terminating with the pure odors (10^{-3}, 10^{-2}, pure). Responses to the filter paper (FP) and paraffin oil (Oil) were also recorded. The time intervals between the conditioning and retention tests and between the two phases are shown.

We used two volatile odors, β-ocimene or OM (purity >90%, Sigma Aldrich, Germany) and phenethyl acetate or PEA (purity 99%, Sigma-Aldrich, Germany), for the first phase of the procedure. These odors were reported as specific for healthy brood (β-ocimene) and larvae infected by the chalkbrood pathogen, *Ascosphaera apis* (phenethyl acetate) and hygienic bees discriminate well between the volatile odors released from the healthy and diseased broods

[59–62]. We used two fatty acids, oleic acid or OA (purity >99%, TCI-Europe NV, Belgium) and linolenic acid or LA (purity >70%, TCI-Europe NV, Belgium) for the second phase of sequential learning that are reported to be involved in the process of kin-discrimination in honeybee [63]. These two odors have similar chemical structures and thus were expected to be harder to differentiate. During the two rounds of differential conditioning, odor contingencies were reversed to understand whether each odor can be learned well either as a CS+ or as a CS- by the bees. Odor stimuli were delivered with a 20 ml plastic syringe containing a filter paper soaked with 10 μl of the respective odor.

Analysis of behavioral data–Quantification of an individualized performance score

A total of 171 honeybees were included in the data analysis. We scored seven behavioral variable to evaluate the olfactory learning performance of the individual honeybees in our assay. Acq-1 and Acq-2: These two variables scored for the rate of CS+ odor learning respectively during the acquisition trials of the first (Acq-1) and the second (Acq-2) differential conditioning. The total number of CRs to the CS+ odor was normalized to the maximum number of CS + responses possible (maximum 5-responses) for an individual bee between the 2nd and the 6th CS+ trial. The PER responses to the CS+ and CS- odors during their respective first conditioning trials were not conditioned and thus were not considered. The scores ranged from 0 to +1 for these two variables. DisCond-1 and DisCond-2: These two variables scored for odor discrimination ability of the bees respectively during the acquisition trials of the first (DisCond-1) and the second (DisCond-2) differential conditioning. They were calculated as the total number of PER to the CS+ stimuli that were not followed by a response to the CS- stimuli, normalized to the maximum number of possible responses (maximum 5-responses) between the 2nd and the 6th acquisition trials. For variable-1 to -4, we only scored the responses of the bees starting from the 2nd conditioning trial of the CS+ and CS- because a response was considered to be a conditioned response only after the 1st conditioning trial. The scores ranged from 0 to +1. DisT-1,-2 and DisT-3,-4: These two variables scored for odor discrimination during the first and second sets of retention tests (DisT-1,-2) of phase-I, and the third and fourth sets of retention tests (DisT-3,-4) of phase-II of the sequential conditioning. The total number of responses to the CS+ stimuli (2 dilutions and pure odors) during the retention test that were not followed by a response to the CS–stimuli (2 dilutions and pure odors) was scored and normalized to the maximum number of possible responses (maximum 3). The scores ranged from 0 to +1. An individualized performance score (P-score) was calculated for each bee by summing the scores of these six behavioral variables. These scores ranged from 0 to +6.

Statistical analysis of behavioral data

Conditioned responses of the bees were analyzed by repeated measures ANOVA followed by the Tukey HSD posthoc test [64–67]. *P*-value was corrected during the posthoc test following the Bonferroni procedure. Wilcoxon matched-pairs test was applied to compare the scores of the behavioral variables for the same set of bees between the two phases of sequential conditioning. The total number of PERs to the CS stimuli during the conditioning and retention tests was compared between the two phases using the G-test. Lilliefors test was performed to understand the nature of distribution of P-scores in the dataset. Mann-Whitney *U* test was used to compare the scores of behavioral variables between the high and low performing bees. Spearman's rank order correlation coefficients were calculated between the scores of the seven variables to understand the nature of associations between them. Hierarchical multiple regression analysis was performed to find out how much of the variability in P-score is explained by

each of the six variables. Statistica version 5.0 (Dell Software, USA) and SPSS version 23 (IBM) were used for statistical analysis and MatLab (MathWorks, USA) for figure making. The analysis of significant gene overlap between the present study and [68] was performed with a χ^2 test (www.socscistatistics.com/tests/chisquare/default2.aspx). The test was applied by considering that the honeybee genome comprises 13440 genes. The differences were considered statistically significant when $p < 0.05$.

Tissue preparation and total RNA extraction

The head capsule of the bee was separated from the rest of the body and a window was cut in the cuticle. The manipulation was performed on a metal slide cooled on dry ice. The head was partially lyophilized in a vacuum chamber at –20˚C for 2 hours and then fixed with Tissue-Tek on a metal slide cooled on dry ice. The brain was exposed by scraping off the hypopharyngeal glands covering the brain and the dorsal part of the central brain containing the mushroom body was recovered and immediately homogenized in 400 μl Trizol (Life Technologies, Schwerte, Germany) in a Teflon glass homogenizer. After phase separation, the aqueous phase containing the total RNA was recovered, mixed in equal volume with 100% ethanol, loaded on a column (RNeasy MinElute Cleanup, Qiagen, Hilden, Germany), treated with DNAse (Qiagen, Hilden, Germany), washed and eluted in water. Extracted total RNA was stored at –80˚C until further use.

Microarray analysis

The methodology described by Gempe and colleagues was applied [43]. The honeybee whole-genome oligonucleotide microarray (Design: UIUC Honey bee oligo 13 K v1, Accession: A-MEXP-755), containing 28,800 oligos that represent 13,440 genes was derived from annotations of the entire honeybee genomic sequence [69]. Ten microliters of each total RNA sample was amplified prior to labelling following the manufacturer's protocol (MessageAmp II aRNA Amplification kit, Ambion). We hybridized 3 μg of each amplified labelled RNA sample to a single microarray slide. The slides were scanned (SureScan Microarry scanner, Agilent, Waldbronn, Germany) and raw hybridization signals were extracted (GenePix Pro 6.0 software, Agilent Technologies). Transcription-level data were processed and analyzed using the LIMMA 2.16 software package (https://www.bioconductor.org/packages/3.3/bioc/html/limma.html). The quality of hybridization was evaluated using the raw expression data from control probes spotted on each slide. Transcription-level data were corrected for background signal ("normexp" function) [70] and intensity-dependent bias was detected ("normalize within arrays" function with the default print-tip LOWESS normalization method) [71]. Finally, the log-transformed expression ratios were calculated and duplicate spots were averaged using the "avedups" function. We used a design matrix that incorporated the P-score, the colony conditions and linear models using the Bayesian fitting option. All microarray data were MIAME-compliant, and the raw data have been deposited in a database (ArrayExpress, EMBL-EBI– https://www.ebi.ac.uk/arrayexpress/experiments/E-MTAB-7909/). Differences in gene transcription that resulted from behavior or from the type of backcross were specified as separate contrasts using linear models. P-values were adjusted for multiple testing with a 5% false discovery rate (FDR). Functional annotation of gene sets that fell into similar categories of gene ontology (GO) terms for molecular processes and biological functions were identified using DAVID (http://david.ncifcrf.gov/) [72, 73] and KEGG [https://www.genome.jp/kegg/], which included an enrichment analysis of GO terms. We used the gene annotations from the UIUC Honey Bee oligo 13 Kv1 annotation file.

RT-PCR

In addition, confirmatory expression analyses were also performed for selected transcripts using RT-qPCRs according to the previously published protocol [74]. Primers are listed in S1 Table and *actin* was chosen as reference gene (TIB Molbiol, Berlin, Germany). Fold change was calculated with the $2^{-\Delta\Delta Ct}$ method. Statistical differences were evaluated using Student's t test for independent measures.

Results

We trained two independent groups of bees in the sequential conditioning paradigm in two phases with each composed of one differential conditioning. Two different pairs of odor were used in each phase; PEA and OM were used for the first phase, LA, and OA for the second phase. To balance odor contingencies, Group-1 (N = 85 bees) bees were trained to discriminate between PEA as the rewarded odor (CS+) and OM as the unrewarded odor (CS-) in the first phase of sequential conditioning while in the second phase, LA was rewarded (CS+) and OA was unrewarded (CS-) (Table 1). Group-2 (N = 86 bees) was conditioned with OM as CS + and PEA as CS-, followed by OA as CS+ and LA as CS-. We first compared the performances of the two groups during the two differential conditioning experiments and found no significant difference between Group-1 and Group-2 when the responses, for each group, were pooled for the two phases, as revealed by a 2 × 2 × 6 (group × stimulus CS+/CS- × trial) ANOVA for repeated measures ($F_{5,3400} = 1.03$, $p > 0.05$). We then pooled the data of all of the four sets of retention tests for each odor-group and compared these groups. The results of a 2 × 2 × 3 (group × stimulus CS+/CS- × trial) repeated measures ANOVA showed no significant interaction effect between group, stimulus and trial ($F_{2,2728} = 1.38$, $p > 0.05$) for the retention tests. Since no significant differences were found in the overall responses of the bees of the two groups to the CS odors during the two differential conditioning and four sets of retention tests, we pooled the data of the two groups for all further analyses.

Learning and memory performance of the honeybees in the sequential conditioning

In the first differential conditioning, bees learned the discrimination task (CS+ vs. CS-) successfully. A 2 × 6 (stimulus × trial) ANOVA for repeated measures produced significant stimulus and trial effects as well as a significant interaction effect ($F_{5,1700} = 99.32$, $p > 0.01$) (Table 3). Similarly, significant stimulus, trial and stimulus × trial effects were also found for the second differential conditioning (Table 2). Hence, the results of the repeated measures ANOVA test demonstrate that bees learned the discrimination task well between the CS+ and the CS–odors during the two differential conditioning (Fig 2A and 2F). The response levels to the CS+ increased across the conditioning trials as the responses to the CS–decreased. The

Table 1. Honeybee groups used for conditioning in the sequential procedure.

Group	1st Phase–Differential Conditioning	2nd Phase–Differential Conditioning
1.	PEA+ vs. OM-	OA+ vs. LA-
2.	OM+ vs. PEA-	LA+ vs. OA-

Two separate groups of bees were trained along the two rounds of differential conditioning in the sequential conditioning paradigm. PEA and OM were used as CS odors in the first conditioning and OA and LA were used in the second conditioning. The contingencies (+: reinforced, -: unreinforced) of the odors were reversed for the two groups of bees during each of the two rounds of differential conditioning to keep the balance in odor contingency.

https://doi.org/10.1371/journal.pone.0304563.t001

Table 2. Results of the repeated measures ANOVA for the two rounds of differential conditioning for the pooled data.

Phase of Sequential Learning	Stimulus Effect	Trial Effect	Trial × Stimulus Effect
1st	$F_{1,340}$ = 164.65, p < 0.01	$F_{5,1700}$ = 12.39, p < 0.01	$F_{5,1700}$ = 99.32, p < 0.01
2nd	$F_{1,340}$ = 30.86, p < 0.01	$F_{5,1700}$ = 3.76, p < 0.01	$F_{5,1700}$ = 81.96, p < 0.01

Fisher statistic values (pooled data) from the repeated measures ANOVA tests are shown for the two differential conditioning. All *p*-values correspond to the stimulus, trial and stimulus × trial effects are significant ($p < 0.01$).

Line graphs are, showing the percent PER to the CS+ (red line) and CS- (blue line) stimuli for the pooled population (N = 171) during the two differential conditioning (A and F) and four sets of retention tests (B, D, G, and I) of the sequential conditioning. Responses to filter paper soaked with paraffin oil (first data point) and filter paper only (second data point) are shown in C, E, H and J. Asterisks denote statistically significant differences ($p < 0.01$) between the PERs to the CS+ and CS–odors. See Table 2 and S2 Table for statistics.

https://doi.org/10.1371/journal.pone.0304563.t002

differences in response levels to the CS+ and CS–increased across the successive trials of the four sets of retention tests, which correlates with the increasing concentrations of the CS odors from the first to the third trial of the retention tests (Fig 2B, 2D, 2G, and 2I, see S2 Table for statistics). During the fourth set of retention tests, bees could not discriminate between the CS + and CS- at their lowest concentration (10^{-3}) (Fig 2I, see S2 Table for statistics). The response levels diminished for the CS–during the retention tests to reach the response levels of the filter paper and paraffin oil (Fig 2C, 2E, 2H, and 2J). No significant difference was found between the responses to the filter paper alone and filter paper soaked with paraffin oil for the two phases of sequential learning (Wilcoxon matched-pairs test: 1st retention test: Z = 1.17, T = 140, $p > 0.05$, 2nd retention test: Z = 0.26, T = 30, $p > 0.05$, 3rd retention test: Z = 0.14, T = 306, $p > 0.05$, 4th retention test: Z = 0.16, T = 182, $p > 0.05$).

Differential responses during the two phases of sequential conditioning

We found that honeybees, which underwent sequential conditioning, learned the odor stimuli but they showed differential responses in the two phases. To investigate the differences in conditioned responses between the first and second phase, we compared the odor discrimination scores quantified for the acquisition trials of the first (DisCond-1) and the second (DisCond-

Table 3. Results of the Mann-Whitney *U* test.

Variable	Comparison Between High and Low Performer Bees
**Acq-1**	(Mean Acq-1_{high} = 0.76, Mean Acq-1_{low} = 0.13)<br>Z-value = 9.94, p < 0.001
**Acq-2**	(Mean Acq-2_{high} = 0.81, Mean Acq-2_{low} = 0.37)<br>Z-value = 7.67, p < 0.001
**DisCond-1**	(Mean DisCond-1_{high} = 0.66, Mean DisCond-1_{low} = 0.13)<br>Z-value = 9.49, p < 0.001
**DisCond-2**	(Mean DisCond-2_{high} = 0.42, Mean DisCond-2_{low} = 0.25)<br>Z-value = 3. 85, p < 0.001
**DisT-1,-2**	(Mean DisT-1,-2_{high} = 0.65, Mean DisT-1,-2_{low} = 0.25)<br>Z-value = 7.54, p < 0.001
**DisT-3,-4**	(Mean DisT-3,-4_{high} = 0.52, Mean DisT-3,-4_{low} = 0.33)<br>Z-value = 4.60, p < 0.001

The results show that high performer bees (N = 118) scored significantly higher than the low performers (N = 53) for all of the six quantified variables related to odor learning, discrimination and memory retention during the two differential conditioning and four sets of retention tests of the sequential conditioning paradigm. The table shows the mean scores for each of the six variables for the two selected groups of honeybees.

https://doi.org/10.1371/journal.pone.0304563.t003

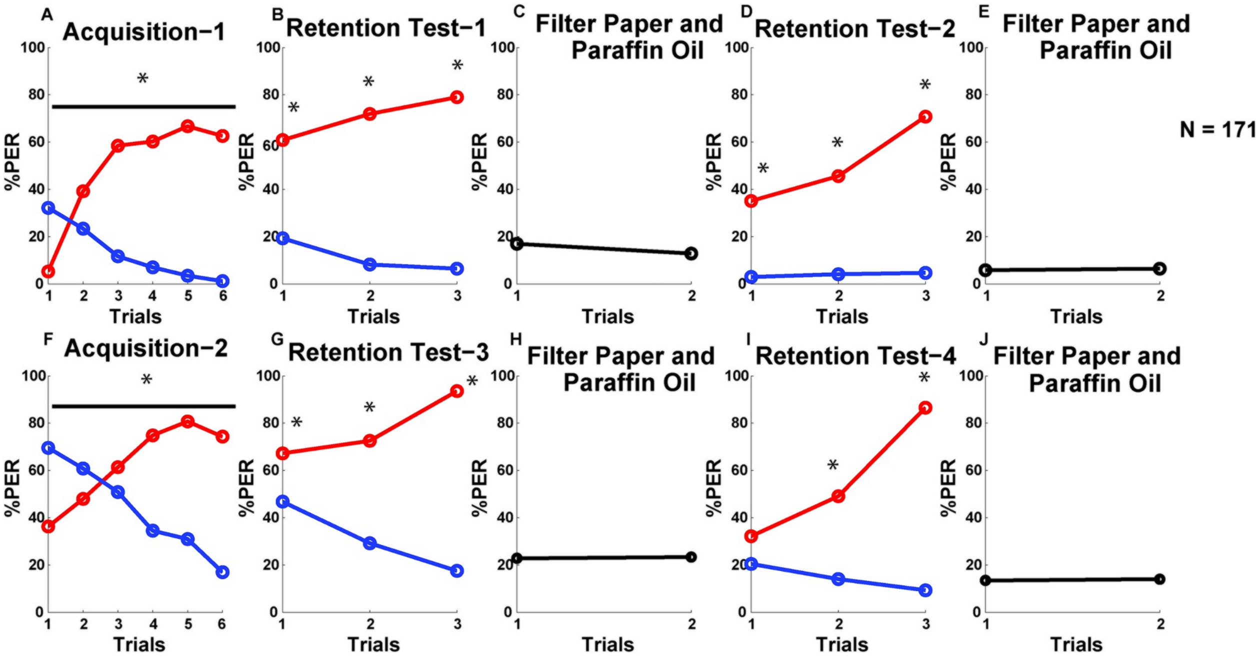

Fig 2. Acquisition and memory retention performances of the bees in the sequential conditioning.

https://doi.org/10.1371/journal.pone.0304563.g002

2) differential conditioning. We found that bees showed significantly higher odor discrimination in the first compared to the second differential conditioning (Wilcoxon matched-pairs test: Z = 4.11, T = 2715, $p < 0.01$) (Fig 3A). This was due to a significantly higher total number

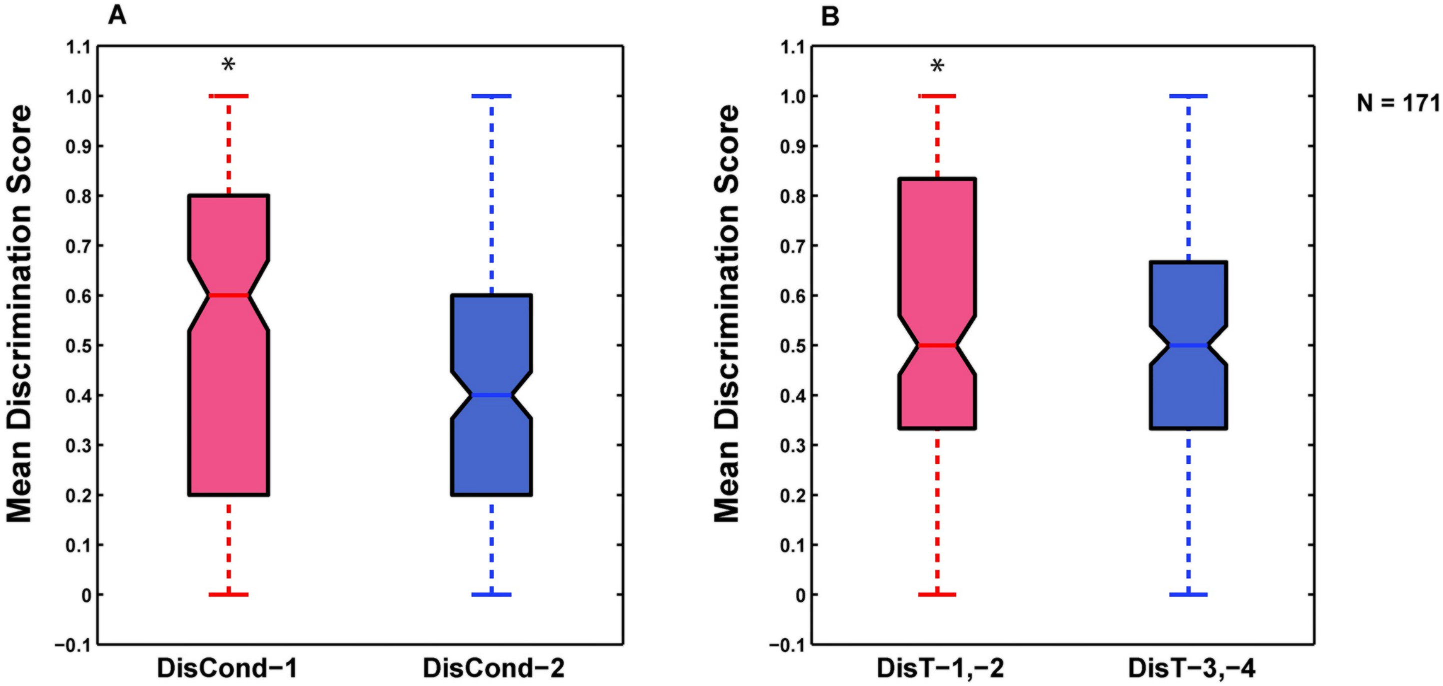

Fig 3. Comparisons of discrimination scores between the differential conditioning and retention tests of the two phases.

https://doi.org/10.1371/journal.pone.0304563.g003

of responses of the bees to the CS- odors during the second compared to the first differential conditioning (first Phase: 491 PERs to the CS+ and 80 PERs to the CS-, second Phase: 580 PERs to the CS+ and 332 PERs to the CS-) (G-test: G = 214.8, df = 1, $p < 0.01$). When we compared odor discrimination scores between the retention tests of the first phase and the second phase of sequential conditioning, we also found a significantly higher odor discrimination during the first and second (DisT-1,-2) sets of retention tests compared to the third and fourth (DisT-3,-4) tests (Wilcoxon matched-pairs test: Z = 2.42, T = 3543, $p < 0.01$) (Fig 3B). This confirms that bees overall discriminated better between the dilutions of the CS+ and CS–odors in the first and second sets of retention test than in the third and fourth sets of tests. Similar to differential conditioning, bees also showed a significantly higher total number of PERs to the CS- odors during the third and fourth sets of retention test compared to the first and second sets of tests (first + second retention tests: 621 PERs to the CS+ and 78 PERs to the CS-, third + fourth retention tests: 686 PERs to the CS+ and 235 PERs to the CS-) (G = 96.63, df = 1, $p < 0.01$). These results show that bees mastered the odor discrimination tasks more efficiently in the first half of the sequential conditioning procedure and generalized more during the second half. The odors used in the second half were also relatively harder to discriminate, especially when diluted (Fig 2 and S2 Table).

The box and whisker plots (displaying the median values, upper and lower quartiles, and the minimum and maximum values of the variables) are showing the distributions of odor discrimination during the first (DisCond-1, red) and the second (DisCond-2, blue) round of differential conditioning (A) and together during the first and second (DisT-1,-2; red), and the third and fourth (DisT-3,-4; blue) sets of retention tests (B). Asterisks denote statistically significant differences ($p < 0.01$). The number of animals is indicated (N).

Classification of the honeybees into various performance categories based on their individual P-scores

Overall performance levels of the individual honeybees in the sequential conditioning procedure are represented by a cumulative performance score (P-score), which is the summation of the six quantified variables (Fig 4). The distribution of P-scores was found to differ significantly from a Gaussian distribution (Lilliefors test: $p < 0.01$) (Fig 4). It varied between 0 and 5.46 with a mean value of 3.12 +/- 1.34 SD. Only 27 bees ($\sim$15.8%) scored $\leq$ 1.78, (< 1-SD of the mean), and 28 bees ($\sim$16.4%) scored $\geq$ 4.46(> 1-SD of the mean) whereas the majority of bees (116 bees, $\sim$67.8%) had scores between 1.78 and 4.46, corresponding to +/- 1-SD of the mean score. We compared the performance levels of the bees with low (P-score<2.6) and high P-scores (P-score> = 2.6), which were categorized as low and high performers (Fig 4). A cut-off P-score of 2.6 was selected based on the lowest score of the high performers and the highest score of the low performers that resulted from the selection of bees for the microarray analysis (Figs 4 and 6). The acquisition and retention test performances were substantially better for the high performer bees than the low performers throughout the two phases of sequential conditioning (Fig 5, see Table 3 for the statistical analysis of between-group performance). The low performer bees showed an overall reduced number of responses to the CS odors throughout the procedure. The most notable effect was observed during the first differential conditioning, with overall very low response levels and no discrimination between the pure odors; as well as between their dilutions during the retention tests-2 and -4 (Fig 5, see S3 Table for the statistical analysis of within-group performance). We applied the Mann-Whitney *U* test and compared the scores of the six variables viz., Acq-1, Acq-2, DisCond-1, DisCond-2, DisT-1,-2, and DisT-3,-4 of between these two categories of bees. The results showed that high performer bees scored significantly higher than the low performers for all of the six variables (Table 3).

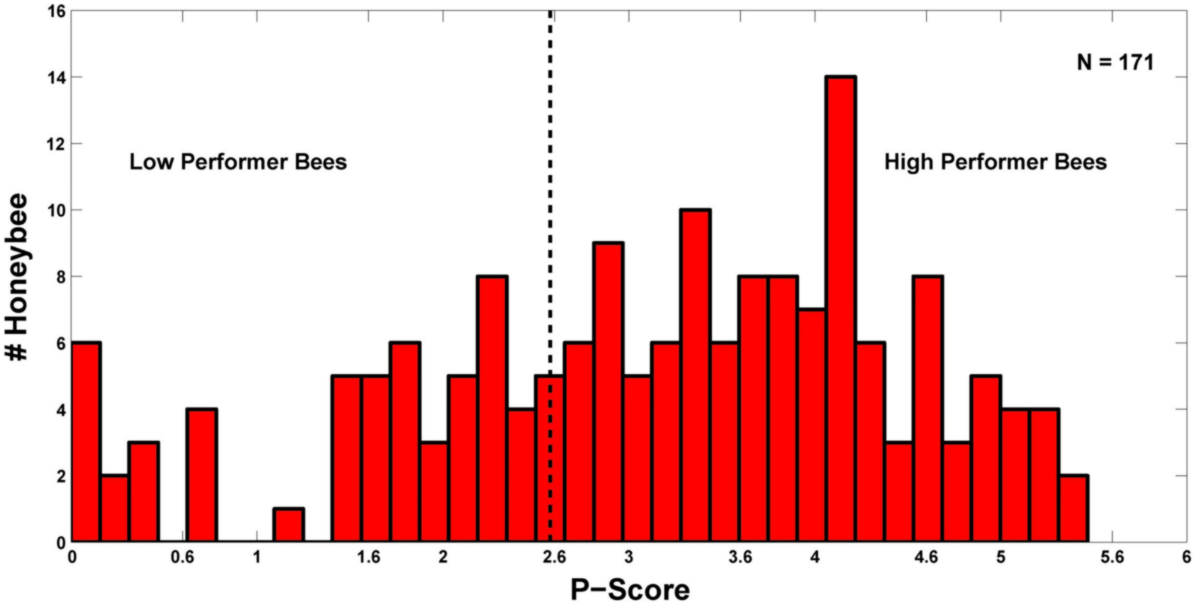

Fig 4. Histogram of P-scores for the pooled data of bees.

https://doi.org/10.1371/journal.pone.0304563.g004

We also separately analyzed the responses of these two groups of bees to filter paper and paraffin oil. We found that high performers also showed significantly higher total number of responses to these stimuli than the low performer bees (G = 24.48, df = 1, $p < 0.001$) (Fig 5). These results demonstrate that high performer bees performed consistently better than the low performers throughout the two phases of sequential learning and retention procedures. The high performers showed early and consistent responses to the CS+ odors and discriminated well between the CS+ and CS- odors during the two differential conditioning. They also successfully discriminated between the different concentrations of the CS+ and CS- odors during the four sets of retention tests. In addition, high performer bees also presented a higher responsiveness to filter paper and paraffin oil stimulation than the low performers.

The bars represent the number of bees constituting all of the P-score categories. The dotted black line represents the threshold in P-score (2.6), used for the selection of low performer (P-score<2.6) and high performer bees (P-score = >2.6). N represents the total number of animals.

Line graphs, show the percent PER to the CS+ (red line) and CS- (blue line) stimuli for the high performer (N = 118) and low performer (N = 53) bees during the two phases of sequential conditioning and retention procedures. The first set of eight subfigures (A to H) represent the PERs to the odors during the first phase. Line graphs of the high and low performers for the same odor trials are given in a pairwise manner, such as Acquisition-1 (High and Low), Retention Test-1 (High and Low), Retention Test-2 (High and Low), Filter Paper and Paraffin Oil (High and Low) to visualize the differences in performance between these two groups of bees. The arrangement of the line graphs is the same for the two groups of bees for the second phase of sequential learning (I to P). Total number of responses to the filter paper soaked with paraffin oil (first data point) and to filter paper only (second data point) are given forthe first (G, H) and the second (O, P) sequential learning phases. The overall responses measured after each retention phases were summed for the high and low performer bees. Repeated measures ANOVA followed by Tukey's HSD posthoc test were performed to analyze the performances of the two groups of bees during the conditioning and retention stages (see S3 Table for

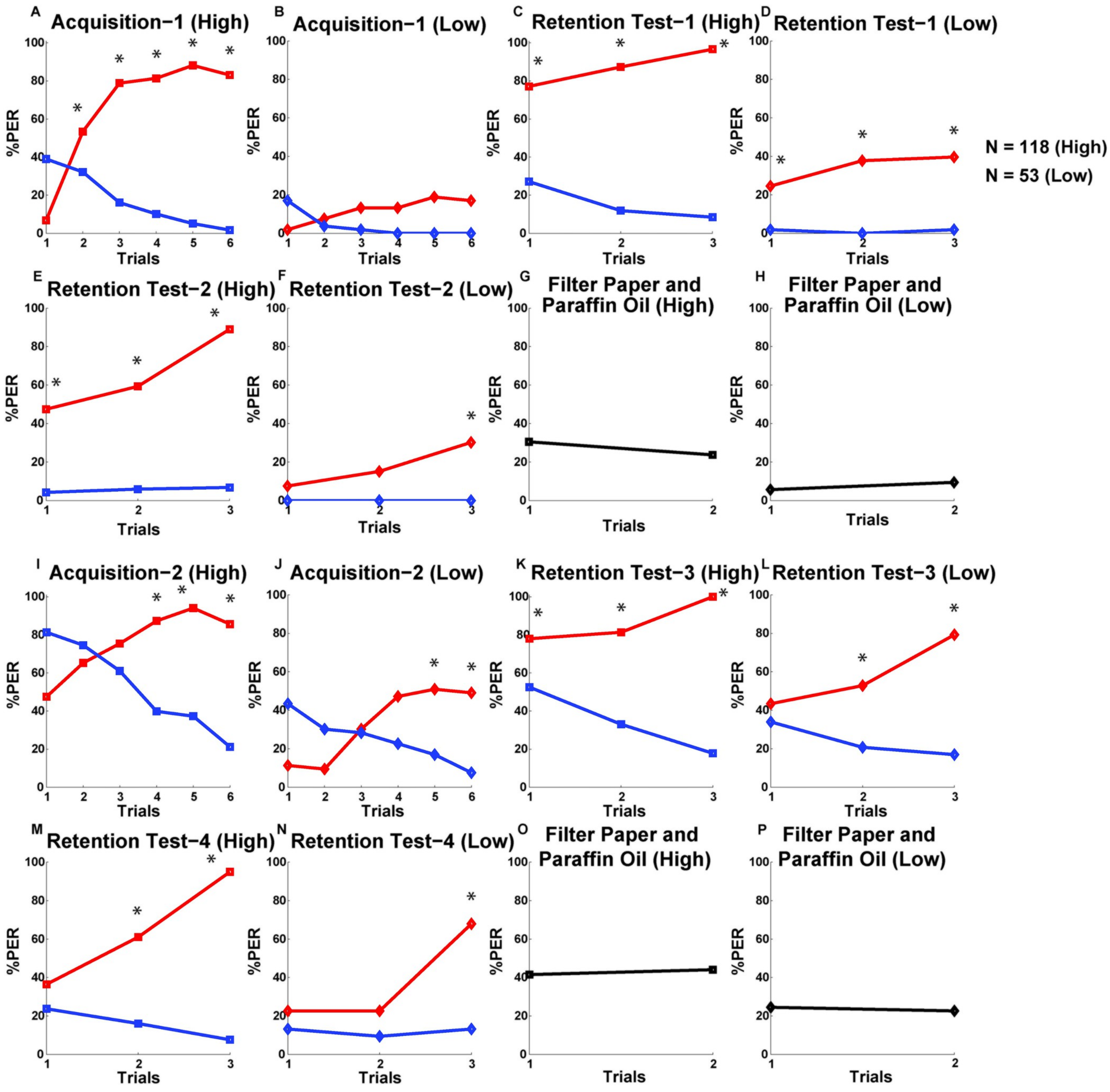

Fig 5. Acquisition and retention performances of the high and low performer bees in the sequential conditioning.

https://doi.org/10.1371/journal.pone.0304563.g005

statistics). Significant differences (after Bonferroni correction of the *p*-value) in responses are represented by asterisks.

A sample of 24 bees (green hexagons with bee-identification numbers) out of the 171 bees (red squares) was selected for the genetic analysis as shown in the scatter plot. Among these 24 bees, 12 were high performers with P-scores ranging between 2.6 and 5.4 and the other 12 were low performers with P-scores ranging between 0 and 2.5.

Predictor analysis of the P-score

Next, we investigated how well the scores of the six quantified variables, related to olfactory learning and memory, predict the P-score. We first looked into the distributions of the six variables and found their distributions to deviate significantly from a Gaussian distribution (Lilliefors test: $p < 0.01$). Thus, we performed nonparametric correlation analysis between these variables and the P-score and found a high correlation (Spearman's $\rho = 0.88$) between the speed of acquisition of the CS+ (Acq-1) and odor discriminability (DisCond-1) during the first phase of differential conditioning (S4 Table). The speed of acquisition of the CS+ and odor discriminability during the first phase also showed high correlation coefficient values with the P-score (both showed a ρ-value of 0.85) (S4 Table). The variable, DisT-1,-2 showed the next highest correlation with the P-score ($\rho = 0.7$) followed by Acq-2 ($\rho = 0.69$) (S4 Table).

To understand the capacity of each of the six variables to predict the P-score, we performed regression analyses to find out how much of the variability in P-score is explained by each of the variables separately. Due to significant correlations between all of the six variables (S4 Table), hierarchical multiple regression analysis was performed to understand the capacities of the individual variables to predict the P-scores. The six regression models, incorporating all of the variables, showed that Acq-1 alone explained the maximum amount of variance (73.3%) in the P-score (Table 4). Like, Acq-1, Acq-2 also explained a significant amount of variance (12.8%) in the P-score (Table 4). Thus, together the rates of acquisition of the CS+ odors during the two phases of sequential conditioning explained 86.1% of the variability in the P-score. The other four variables also contribute to the variance of the P-score: DisCond-1 (3.5%), DisCond-2 (3.7%), DisT-1,-2 (4.2%) and DisT-3,-4 (2.5%) (Table 4). The six variables significantly increased the predictive capacities of the subsequent regression models (Table 4) and together they explained 100% variance in the P-score (shown by the R^2-value of Model-6) because P-scores are unweighted summations of these six variables (Table 4). These results illustrated that the rate of learning of the CS+ odors during the first phase of differential conditioning or Acq-1 had the highest predictive capacity for the P-score.

Microarray analysis of high and low performer bees

Twenty-four bees were selected for the microarray analysis based on their performance during the behavioral tests, the colony and the date of the experiment (Fig 6 and Table 5). Bees from

Table 4. Results of the hierarchical multiple regression analysis.

Variable	R^2	R^2-Change	Model & Variables	Model Statistics (ANOVA)	Standardized Regression Coefficient (β)
**Acq-1**	0.733	0.733	Model-1 (Acq-1)	$F_{(1,169)} = 463.82$, $p < 0.001$	0.269
**Acq-2**	0.861	0.128	Model-2 (Acq-1, Acq-2)	$F_{(2,168)} = 522.13$, $p < 0.001$	0.239
**DisCond-1**	0.896	0.035	Model-3 (Acq-1, Acq-2, DisCond-1)	$F_{(3,167)} = 480.36$, $p < 0.001$	0.244
**DisCond-2**	0.934	0.037	Model-4 (Acq-1, Acq-2, DisCond-1, DisCond-2)	$F_{(4,166)} = 582.92$, $p < 0.001$	0.197
**DisT-1,-2**	0.975	0.042	Model-5 (Acq-1, Acq-2, DisCond-1, DisCond-2, DisT-1,-2)	$F_{(5,165)} = 1309.8$, $p < 0.001$	0.227
**DisT-3,-4**	1.0	0.025	Model-6 (Acq-1, Acq-2, DisCond-1, DisCond-2, DisT-1,-2, DisT-3,-4)	$F_{(6,164)}$ = , p	0.181

The results show the values of R^2, changes in R^2, the consecutive regression models with their variables, corresponding model statistics, and the β–values for the six variables. Acq-1 explains the highest, 73.3% variance in the P-score. Together the six variables explained 100% variance in the P-score (shown by the R^2 value for Model-6). No F-statistic and *p*-values are shown for DisT-3,-4 by SPSS. For model-1 to -5, statistics are found significant ($p < 0.01$).

https://doi.org/10.1371/journal.pone.0304563.t004

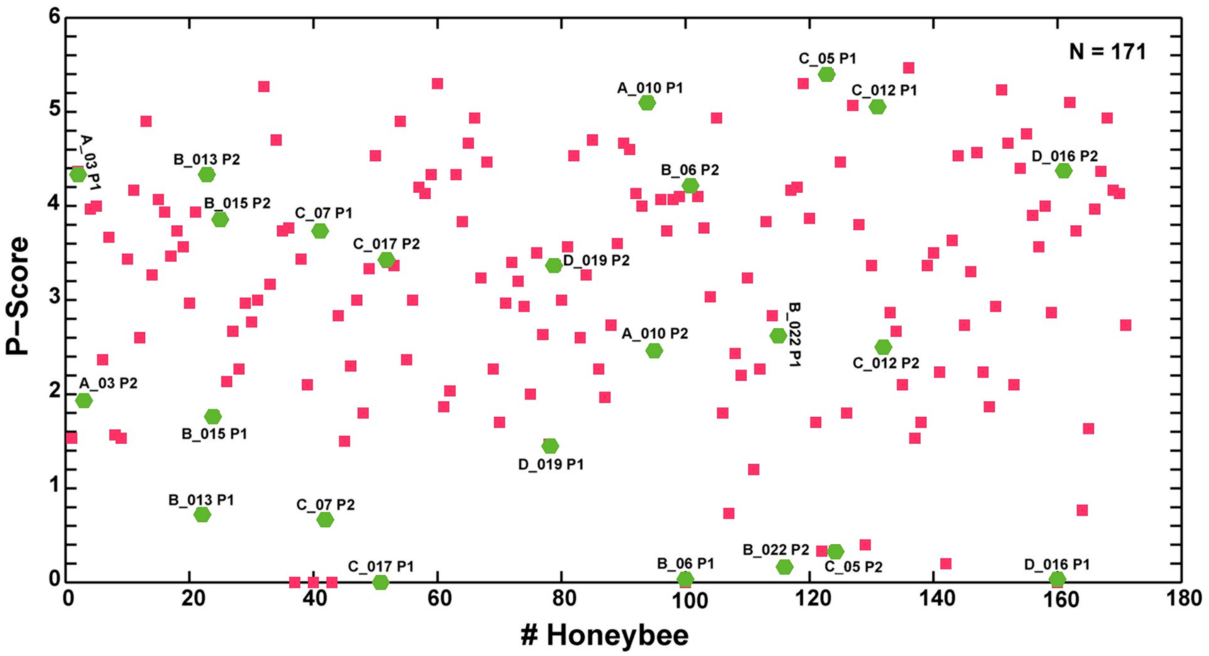

Fig 6. Scatter plot of the P-score for 171 bees.

https://doi.org/10.1371/journal.pone.0304563.g006

the colonies 67, 98, 73 and 299 were used for this analysis. The total RNAs extracted from the mushroom bodies were processed to generate the microarray probes. Two sets of probes were generated for each bee, one labelled with Cy5 and the other with Cy3. In this manner, each animal was analyzed twice by swapping colors on each array, to reduce dye-related artefacts. The last bee of the list was analyzed with the first one, thus the analysis scheme was designed to build a loop. High performer bees had P-scores ranging between 2.6 and 5.4 and low performers between 0 and 2.5 (Fig 6 and Table 5). Eight bees each from the colonies 98 and 73 and four bees each from the colonies 67 and 299 were analyzed (Table 5). The cut-off value of 2.6 is subjective between high and low performers. Indeed, the selection of bees for the gene expression analysis was performed by keeping the colony and seasonal effects, which are other sources of variation in gene expression, as low as possible. To this end, high and low performers bees selected from the same colony at the same time of the year were compared on the one array slide (Fig 6 and Table 5). To reduce the colony effects further on the identification of genes, high performer bees with P-scores ranging from 3.9 to 5.3 from the four colonies were also compared on 12 arrays (S5 Table). In addition, each colony was also analyzed with three bees collected from July to October 2010 and dye swap loop design was applied (S5 Table).

The microarray analysis of the behavioral design generated a list of 687 genes and the analysis of colony specific differences (genotype design) generated a list of 265 candidates. The behavioral list was sorted by removing 32 gene candidates, which were also present in the genotype list, reducing the behavioral list to 652 candidates that were differentially expressed between the high and low performer bees with adjusted *p*-level varying between 0.0025 and 2.9×10^{-7} (S6 Table). Most of the identified genes only showed slight differences in expression levels. The expression of 371 genes was reduced in the high performer bees, varying between 0.75× and 0.95× and the expression of 281 genes was increased between 1.05× and 2.46×. A list of 45 identified genes, including the candidates with the highest changes in expression levels, is presented in Table 6. They potentially encode proteins implicated in diverse biological

Table 5. Selection of the pairs of bees for behavioral analysis.

P-Score	Bee	Colony	Month	Dye
0	C_017 P1	98	September	Cy5
				Cy3
3.4	C_017 P2	98	September	Cy5
				Cy3
0.66	C_07 P2	98	August	Cy5
				Cy3
3.73	C_07 P1	98	August	Cy5
				Cy3
0.33	C_05 P2	98	August	Cy5
				Cy3
5.4	C_05 P1	98	August	Cy5
				Cy3
2.5	C_012 P2	98	August	Cy5
				Cy3
5.06	C_012 P1	98	August	Cy5
				Cy3
2.46	A_010 P2	67	August	Cy5
				Cy3
5.1	A_010 P1	67	August	Cy5
				Cy3
1.93	A_03 P2	67	July	Cy5
				Cy3
4.36	A_03P1	67	July	Cy5
				Cy3
0	B_06 P1	73	August	Cy5
				Cy3
4.2	B_06 P2	73	August	Cy5
				Cy3
1.76	B_015 P1	73	September	Cy5
				Cy3
3.86	B_015 P2	73	September	Cy5
				Cy3
0.73	B_013 P1	73	September	Cy5
				Cy3
4.33	B_013 P2	73	September	Cy5
				Cy3
0.16	B_022 P2	73	September	Cy5
				Cy3
2.63	B_022 P1	73	September	Cy5
				Cy3
0	D_016 P1	299	September	Cy5
				Cy3
4.36	D_016 P2	299	September	Cy5
				Cy3
1.46	D_019 P1	299	September	Cy5
				Cy3
3.36	D_019 P2	299	September	Cy5
				Cy3

Selection of the pairs of bees from the four colonies (Colony), conditioned together (on the same day) in the different months of the experimental year (Month), for the behavioral analysis was based on their high and low P-scores. The column 'Bee' is showing the identification number of the bees. Processed total RNAs were labeled with Cy3 and Cy5 for microarray analysis.

https://doi.org/10.1371/journal.pone.0304563.t005

Table 6. List of genes, from the sorted behavioral list, that showed the strongest changes in expression levels between the high and low performers.

Sr. No.	Quantitative PCR	Honeybee mRNAs	Fold Change	Average Expression	Adjusted p-Value
1.		GB12452	0.75	11.42	5.5E-05
2.	**RT-qPCR**	GB11630 Excitatory amino acid transporter 2	0.82	13.12	9.8E-06
3.		GB14954 metabotropic GABA-B receptor subtype 1	0.85	10.57	9.0E-07
4.		GB13493 blue-sensitive opsin (Blop)	0.86	14.51	1.2E-04
5.	**RT-qPCR**	GB15141 Dscam	0.86	9.80	1.3E-03
6.		NM_001011594 G-protein coupled receptor (Tyr1)	0.88	12.01	4.2E-05
7.		XM_392481.6 Apis mellifera peroxidase	0.88	12.43	6.9E-04
8.		EST122 elongation factor-1alpha F2 gene	0.89	14.01	2.6E-04
9.		GB15633 Calmodulin	0.89	14.73	4.6E-04
10.		GB11423 casein kinase II, alpha 1 polypeptide	0.90	10.82	5.5E-06
11.	**RT-qPCR**	GB19379 SUMO (ubiquitin-related) homolog family member	0.91	11.68	1.6E-04
12.	**RT-qPCR**	NM_001011629 putative transcription factor mblk-1	0.92	12.74	3.9E-05
13.		CG14998 Drosophila melanogaster ensconsin	0.92	12.11	5.2E-04
14.		GB30031 dopamine receptor, D1 (Dop1)	0.92	11.66	1.8E-03
15.		GB20129 minibrain	0.92	10.95	1.5E-03
16.	**RT-qPCR**	GB14853 atlastin	0.94	12.10	2.9E-04
17.	**RT-qPCR**	NM_001011629 transcription factor mblk-1 (Mblk-1)	0.94	14.01	1.9E-03
18.		GB16449 phosphatidylinositol 4-kinase type II	1.07	10.94	1.1E-03
19.		GB11336 Putative proteasome inhibitor	1.07	13.04	1.7E-04
20.		GB15333 adenylate kinase 3	1.07	11.82	4.7E-04
21.		GB15168 VAMP-associated protein A	1.07	12.91	7.9E-05
22.		GB12827 26S proteasome non-ATPase regulatory subunit 3	1.08	10.10	6.3E-04
23.		GB11560 Growth hormone-inducible transmembrane protein	1.09	12.70	2.0E-03
24.	**RT-qPCR**	GB17254 nicotinic acetylcholine Apisa7-2 subunit	1.09	12.83	2.2E-03
25.	**RT-qPCR**	GB15359 alpha isoform of regulatory subunit A. protein phosphatase 2	1.10	11.90	2.1E-04
26.		GB19754 Juvenile hormone-inducible protein 26	1.10	11.48	1.9E-04
27.		GB19372 GABA neurotransmitter transporter-1A	1.11	13.39	1.1E-03
28.		GB19007 tyrosine kinase	1.11	13.62	2.0E-03
29.		GB11373 Ras-related protein Rac1	1.11	10.72	1.9E-04
30.		GB16567 TWiK family of potassium channels family member (Twk-39)	1.14	10.20	7.5E-04
31.		GB14433 syntaxin 7	1.14	10.35	1.4E-04
32.		GB19296 Rab-protein 2	1.14	11.48	9.4E-04
33.		XM_001122661 Glutamate oxaloacetate transaminase 1	1.16	11.36	1.0E-03
34.		GB10489 Na/K-transporting ATPase subunit beta-2	1.20	11.71	1.5E-04
35.		NW_001253371.1 miRNA HC_mir-34	1.20	12.78	5.0E-04
36.		GB15139 malvolio (Mvl)	1.20	11.36	2.5E-06
37.		GB11820 inositol polyphosphate-1-phosphatase	1.20	12.29	2.8E-04
38.		GB18844 Glutamate oxaloacetate transaminase 1	1.20	10.39	3.2E-05
39.		GB15813 odor binding protein 8 (Obp-8)	1.21	10.15	3.9E-04
40.		GB16853 Nedd4	1.22	12.83	1.2E-03
41.		GB11213 FKBP12-rapamycin complex-associated protein	1.23	10.65	2.6E-04
42.		GB13939 neuroligin	1.37	11.01	1.3E-03
43.		GB18312 alpha-amylase	1.90	13.16	3.5E-04
44.		GB19418 glucose oxidase	2.20	12.67	1.8E-06
45.		GB16382 major royal jelly protein 5 (Mrjp5)	2.46	12.67	4.1E-04

A selection of 45-identified genes from the sorted behavioral list that includes candidates with the strongest changes in expression levels between the high and low performers. The table shows the accession numbers and identity of the mRNAs deduced from the genome annotation (Honeybee mRNAs), the change in expression levels between the high and low performers (Fold Change), the averaged expression levels measured on the microarray and the adjusted *p*-values denoting significant changes in expression levels between the high and low performers, calculated from the microarray analysis. Selected transcripts were further validated by RT-qPCR (S1 Fig)

https://doi.org/10.1371/journal.pone.0304563.t006

functions, including neurotransmitter receptors for dopamine, tyramine, GABA and acetylcholine, proteins implicated in the synaptic release of neurotransmitters (VAMP, syntaxin, neuroligin) and amino-acid transporters (GABA, excitatory amino-acid transporters). Several candidates were implicated in cellular processes important for memory formation (proteasome complex, TOR pathway, protein translation) and in development (the transcription factor *mblk-1*, *minibrain*). Other interesting candidates, including calmodulin, kinases, and phosphatases were also implicated in messenger pathways important for memory formation.

Interestingly, genes, which are regulated by growth hormone or juvenile hormone and *malvolio*, a gene implicated in the division of labor, also showed increased expressions in the high performer bees. Surprisingly, RNAs known to be absent from the mushroom body were also found in the list. The blue opsin gene, normally expressed in the visual system probably originated from a contamination with the ocellar tract. In addition, genes including, α-amylase, major royal jelly protein 5 (*mrjp5*) and glucose oxidase also showed the strongest differential expression with their expression levels doubled in the high performer bees. These RNAs probably originated from a contamination with the hypopharyngeal glands, , which are adjacent to the MB.

Some genes of the whole genome honeybee microarray could be linked to the orthologues of the *Drosophila* Flybase. This way, a list of 418 Flybase entries were generated from the sorted honeybee behavioral list (S6 Table). KEGG Automatic Annotation Server (KAAS) analysis revealed 600 enriched terms, including those related to sugar metabolism (e.g. pentose, fructose, galactose and sucrose), lipids metabolism (e.g. sphingolipid, arachidonic acid, linoleic acid), amino acid and nucleic acid metabolism (e.g. spliceosome, RNA transport, Ribosome) (S7 Table). The results also highlighted terms directly related to classical neurotransmitter systems (GABA, acetylcholine, glutamate, dopamine serotonin), olfactory transduction, axon guidance, synaptic transmission (e.g. SNARE, Tight junction, GAP junction), the proteasome complex and several signaling pathways (e.g. Ras, MAPK, Ca^{2+}, cAMP, cGMP, mTor). Many terms related to hormonal control (e.g. insulin, oxytocin, prolactin, adrenergic) and food processing (salivary-, gastric acid-, pancreatic-secretion, etc.) were also described. Several terms not directly related to the neuronal metabolic pathways underlying memory processes were also highlighted (e.g. immune response, longevity).

The behavioral gene list was further submitted to DAVID and the functional annotation chart revealed 76 statistically significant terms. Some of these were related to microtubule-associated complex, mitochondria, neurogenesis, plasma membrane, splicing, GTP activity, oxidoreduction and nucleic acid metabolism (S8 Table). The first eleven clusters, presenting enrichment scores between 1.45 and 0.81, highlighted terms related to small GTP-binding protein domain, mitochondrion, PDZ domain, glycoside hydrolase, α/β hydrolase, spliceosome, protein phosphatase, actin cytoskeleton, neuroblast, small GTPase superfamily and lipid metabolism (S8 Table).

Validation of gene candidates by RT-PCR

Two low and two high performer bees from each of the four colonies (S9 Table) were selected for the validation of nine selected genes by reverse transcription quantitative real time PCR technique (RT-qPCR) (Table 6). Low performers had scores varying between 0 and 3.33 and high performers between 3.8 and 5.4. These bees were collected between July to October 2010. None of the tested genes showed significant difference between the high and the low performer groups (Student t-test, $p > 0.05$) (S1 Fig).

Discussion

A deeper understanding of the variations in individual behavior within species is attracting more attention owing to their significant contributions to the ecological traits of the species and their evolution [9, 75, 76]. Heterogeneity in behavioral types or personality, which is related to the difference in cognitive ability of individuals, is a heritable feature in animals that controls important life-history traits, such as food intake, detecting prey, predators and mates, labor division, ability to respond better to the environmental change, and overall fitness [76–80]. Honeybees show individual variability in behavior, cognitive task specialization and consistency in learning proficiency across learning paradigms and stimulus modality [81–85]. Furthermore, an early study in honeybee showed substantial genetic contribution to the variance observed in learning phenotype, which supports the heritability of among-individual difference in cognitive ability [22]. Hence, this species is an appropriate model system to study the nature of heterogeneity in cognitive abilities. In honeybee, it is already established that the group-averaged analysis of the learning data impedes the visualization of this heterogeneity in individual's learning, viz. the learning rate, final level of learning, and the strength of retained memory [30, 31]. Previously, response latency has been described to have the potential to underlie the heterogeneity in learning dynamics of the individual bees, which was explained by a heterogeneous Rescorla-Wagner model [30, 31]. However, these analyses could not capture a clear picture of the identity of a salient behavioral feature(s) that determines the individualistic variability in learning ability in the honeybee populations. Here, we addressed this issue through a complex form of olfactory learning protocol and analyzed the data to understand the contributions of different learning-related features to the overall performance levels of the individuals. Further, correlations between learning performance and the expression patterns of learning-related genes in the brains of these individual honeybees were evaluated.

Methodological consideration

We trained the honeybees in a sequential olfactory conditioning protocol in which the animals received two consecutive stages of differential training with two different pairs of odors with each differential conditioning stage followed by two sets of memory retention tests in which we applied several dilutions of the rewarded (CS+) and unrewarded (CS-) odors. The protocol was specifically designed to test the responses of the individual bees rigorously for a longer period to find out the strength in their learned responses. The tasks of discrimination between the CS+ and CS- odors during the two stages of conditioning were easier compared to the four sets of retention tests because of the use of pure odors instead of their dilutions during the conditioning; thus, the 'Aha' effect of learning led to clear separation in responses of the bees to the odors [86, 87]. Individual honeybees however had to perform consistently well throughout the procedure to reach a higher cumulative performance score (P-score) or they failed at different time points to score lower. This way we were able to distinguish between the low and high levels of olfactory learning performers present in the natural populations. Bees from all four colonies learned the odor stimuli during the conditioning and discriminated well in the retention tests. However, the discrimination task was more difficult when the cuticular volatile fatty acids (OA and LA) were used compared to the brood volatiles (OM and PEA). We speculate that learning of differential contingencies of these two odors is difficult in the perceptual space of honeybees owing to the structural similarities of these two compounds (both are 18-carbon, unsaturated essential fatty acids) [88]. This could be a prime reason for the bees to generalize more between these two odors during the second half of the procedure in addition to the possible involvements of the non-associative components, like sensitization and hunger due to prolong testing of the animals for more than 6 hours with repeated sucrose

stimulations. The associative components together with the non-associative counterparts make our procedure more stringent and specific in discriminating between the low and high levels of learning performances. We quantified the speed of learning of the CS+ odors and the levels of odor discriminability both during the two sets of conditioning and four sets of retention tests and investigated whether there is a single salient feature that persistently contributes to the overall learning performance of the individual bees. This could be considered as an important feature that controls the heterogeneity in learning dynamics among the individuals of the natural populations. There are other complex olfactory learning protocols that were previously applied on the honeybees [66, 89] but that we did not use because these protocols were either not recording the bee's responses for a longer period or they incorporated extinction components in learning, both of which were avoided. It is conceivable that an extinction-learning component prevailed in our sequential conditioning design as we performed two consecutive sets of retention tests after each of the two differential conditionings. Indeed, bees responded less to the highest dilution of the CS+ odors (10^{-3}) in the second compared to the first set of retention test (in the paired-retention tests). However, no difference in memory recall was found for the training concentrations of the CS+ odors in the two consecutive sets of retention tests. Thus, we conclude that no extinction learning took place in our experimental design in which we offered no time gap between the consecutive sets of retention tests.

Speed of learning explains the heterogeneity in learning and memory performance of the individual honeybees

Very few studies have systematically investigated the nature of heterogeneity or individualistic variation in the learning ability of honeybees. Tait and colleagues have reported individualistic variation in honeybee in the domains of olfactory and navigational learning as well as maneuverability performance; however, the authors did not conclude anything with regard to the source of variation in the learning behavior of individuals [23]. Our analysis clearly demonstrates a large heterogeneity in the learning ability of the individual honeybees, which is structured with a continuous range of performance. Further, the variation in the speed of odor learning appears as the major component for this heterogeneity. We found a non-normal distribution of the P-score in our experimental population, which may be due to low sample size. Interestingly, normal distribution was reported for several cognitive capacities in human populations [90]. The distribution of P-score in our study might reflect different degrees of specialization for particular learning modalities among the honeybees, which are at different developmental stages and are involved in different tasks [82, 91, 92]. Indeed, variation among the individuals can emanate from the development of specific experience-dependent biases and thus, differences in physiologies to detect, learn and respond to particular external stimuli [93].

The large heterogeneity in the learning dynamics of individual honeybees as seen in ours as well as in the previously generated learning data sets [30, 31] can probably be attributed to several factors, such as stimulus sensitivity, speed of learning, and memory retention capacity. We found that high performer bees performed consistently well throughout the 56 trials of odor conditioning and retention tests compared to the low performers. They have mastered the tasks of odor discrimination during the conditioning and showed specific responses to the rewarded odors during the retention tests as well as responded more to the air puff stimulations without any odors (filter paper only or with paraffin oil) than the low performers (Fig 5). Importantly, the responses to the filter paper also varied between individual bees and thus contributed to the individual variability. These results together with higher responses of the high performers compared to the low ones to the highest CS+ dilution (10^{-3}) during the retention tests indicate possible higher olfactory sensitivity among the high performers.

The feature that correlated most strongly with the P-score was a higher acquisition rate during the first phase of conditioning. The speed of learning of the CS+ odor during the first differential conditioning (Acq-1) best predicted the cumulative performance of the individuals among all six quantified variables. Individuals with higher speed of CS+ odor learning not only discriminated the odors well during the first phase of differential conditioning, they also had higher retention scores and higher odor sensitivity. Although the honeybees also performed successfully in the second differential conditioning phase, only the speed of acquisition during the second differential conditioning (Acq-2) correlated strongly with the speed of acquisition during the first differential phase. Acq-2 was also the second strongest predictor of the cumulative score. The discrimination index during the second acquisition phase (DisCond-2) and the discrimination during the second retention test (DisT-3,-4) correlated with Acq1. These variables are more influenced by the capability of an individual to adapt its behavior to new conditions and it adds an independent value to the selection of high performers.

Fast and slow learning is elaborately discussed in behavioral ecology in the context of behaviors that involve speed-accuracy trade-off. A fast learner can take more risk to gain more only for a short-term and even perhaps lacks accuracy, whereas a slow learner has better accuracy and it can perform the duty more safely with the notion for a long-term gain [75, 94]. The advantage of having slow learners has also been reported in a study on bumblebees where fast learning of visual information was found to be associated with lower number of days of foraging compared to the slow learning bumblebees. This suggests that superior cognitive abilities may not be beneficial to build the reserve of the colonies [95].

We were unable to test the change of conditioned responses over time shortly after learning [96]. However, our results clearly show that bees, which started responding early to the CS + odor compared to the late responders during the first conditioning, showed stronger and more specific odor memories in the short-term recall tests. The findings on memory specificity are contradictory to the findings of Pamir and colleagues [31] who used an absolute training procedure and found no difference in the specificity of odor memories between the early and late responders. We assume that this difference in results could be due to the differences in our conditioning methods and odor stimuli used, because the present sampling method is more controlled. We thus surmise from our results that the mechanisms underlying the speed of learning of odor stimuli are correlated to those controlling their discrimination and consolidations of memories.

Genetic analysis in the mushroom body area of the high and low performers

An analysis of differences in gene expression in the mushroom body (MB) region of the low and high performers was performed holistically using microarray. The analysis identified 652 differentially expressed genes; most of them were characterized by small differences in expression levels between the high and low performer bees. This indicates that many genes each with small amplitude effects may contribute to the high performer phenotype. We selected nine RNAs from the transcript list originally generated by the microarray method to further verify their abundance by RT-qPCR. The presence of all nine transcripts could be confirmed by RT-qPCR, but no significant differences in abundance was detected in the high and low performer groups by this technique (S1 Fig). These results illustrate that behavioral traits are influenced by small changes in expression levels of multiple genes [97]. This particularity is also a feature described for neurological disorders [98, 99].

The KEGG and DAVIDD analyses revealed similar terms. Nervous system specific terms were related to plasma membrane and synaptic transmission, illustrated by neurotransmitter

receptors (GABA, dopamine, octopamine, acetylcholine, glycine and serotonin) and to synaptic components related to neurotransmitter release (e.g. SNAREs). Terms related to LTP, LTD, and neurogenesis were also revealed as well as terms related to olfaction, taste and photo-transmission. The latter might indicate contamination with tissues adjacent to the MB. Indeed, some of the terms can also be related to increased metabolic activity, like sugar metabolism, or to mitochondria and cellular respiration. The analysis of the gene list also indicates that the MB dissections were contaminated with hypopharyngeal glands as we identified higher levels of the major royal jelly protein 5 gene (*mrjp5*) in the high performers. Indeed, all *mrjp*s are produced in the hypopharyngeal glands except for *mrjp3* that is produced in the Kenyon cells of the MB of nurse bees [100–102]. Equally, α-amylase and glucose oxidase, both of which were overexpressed in the high performers, are also produced in the hypopharyngeal glands [103, 104]. However, several intracellular signaling cascades, known to be implicated in learning and memory, were also highlighted, including several second messenger pathways (TOR signaling and the ubiquitin proteasome complex). These pathways regulate, among others, translation- and transcription-dependent processes that are important in memory processes and are highlighted in different lists. However, these processes are not restricted to neurons and the increased RNA abundances might have arisen from the activities in adjacent tissues.

The hypopharyngeal glands are important for food processing in honeybees [105–108]. The genes *mrjp*1-4 and *7* are more active in the nurse bees compared to the foragers [101, 102]. In foragers, α-amylase and glucose oxidase are associated with the conversion of nectar to honey [103, 104]. The change in expression of these enzymes is dependent on the age and the task. Interestingly, *malvolio*, a protein influencing labor division in honeybees, was also overexpressed in the high performers [109, 110]. This manganese transporter was discovered in *Drosophila* and is known to influence sucrose responsiveness [111]. Thus, we hypothesize that the high performer bees are in the transition from nursing to foraging activities, which is supported by the highlighted genes and terms related to hormonal pathways. It is also known that immunity changes during this transition [104, 112] and terms related to immune response were also highlighted in the gene ontology analysis.

In a previous study, Whitfield [68] and colleagues analyzed behavioral maturation in the honeybee by comparing the gene expression profiles in the brains of bees in different settings; nurses and foragers of specific ages compared in crossed conditions as well as nurses treated with different chemicals, described for their roles in the onset of foraging, Methoprene (an analogue of juvenile hormone), manganese and cGMP. Both settings were reported to associate with the molecular pathways of *malvolio* and *foraging*. However, cAMP was found to play no role in the onset of foraging [68]. We compared our gene list to the gene lists of them. A comparison of our list of genes with these gene sets, characterized by a significance level < 0.05, did not reveal any significant overlap. By restricting the gene sets to candidates, characterized by a significance level < 0.001, significant effects were described (Table 7). A significant correlation was found for Methoprene treatment, a condition also implicated in the transition from nursing to foraging. However, there was no significant overlap with manganese or cGMP treatment or when comparing 17 days old foragers and nurses (d17F/d17), which are conditions specific for foragers. Significant overlap was found with gene sets specific for juveniles, which are still active with hive duties but able to perform the transition from nursing to foraging (d4/d8 and d12/d17). This indicates that high performers belong to a group of young bees making the transition to foraging activities. The animals were caught at the end of the afternoon, when mostly foragers perform outbound flights. Thus, these bees might correspond to young individuals beginning their foraging activity. It is known that young foragers and precocious forager honeybees learn olfactory stimuli better than the nurse bees [82, 113]. However, less is known about differences between young and old foragers.

Table 7. Comparative analysis of the list of genes between this study and the one published by Whitfield and colleagues in 2006.

Treatment	Methoprene	Manganese	cGMP	cAMP	cAMP/cGMP
Total Differentially Expressed Genes	481	509	911	129	327
Gene Overlap	24	11	28	7	10
c^2	13.8566	0.1564	1.7335	4.9555	0.5665
p-Value	0.000197	0.692517	0.187961	0.026008	0.451641
Age	d1/d4	d4/d8	d8/d12	d12/d17	
Total Differentially Expressed Genes	2613	1260	342	160	
Gene Overlap	69	44	11	8	
c^2	0.6338	6.6813	0.9272	4.5344	
p-Value	0.425968	0.009743	0.33559	0.03322	
Age	d17/d1	[d8,d12,d17]/d1	d17F/[d8,d12,d17]	d17F/D17	
Total Differentially Expressed Genes	3745	3563	3575	2414	
Gene Overlap	101	97	94	62	
c^2	1.6149	1.8049	0.8545	.2534	
p-Value	0.203798	0.179123	0.355274	0.614713	

Comparison of the gene list with adjusted p value < 0.001, described in this study (n = 326) and in the study of Whitfield and colleagues (2006) [69]. The number of differentially expressed genes identified for specific conditions are presented (Total differentially expressed genes). There were treatments with Methoprene, Manganese, cGMP, cAMP, and cAMP vs cGMP. Age effects were also considered in the nurse bees of different ages (days, d). Seventeen days old nurses and foragers were also compared (d17F/d17). We evaluated if the number of overlapping genes between the sets identified in this study and those of Whitfield (Gene overlap) was random with a χ^2 test. Significant differences have a p value < 0.05.

https://doi.org/10.1371/journal.pone.0304563.t007

Only a few studies addressed this question [114, 115]. The correlation with the gene set specific for the cAMP second messenger pathway can explain the higher sensitivity of the high performer bees compared to their low performing counterparts. Indeed, this second messenger pathway is associated to modulatory neurotransmission mediated by the biogenic amines, including serotonin, octopamine, dopamine, and tyramine that are implicated in arousal, sensitivity, and learning performances [82, 116, 117]. The cAMP second messenger pathway is linked to some of these neurotransmitter systems, which might explain why the high performer bees are responding more to olfactory stimulation with filter paper and paraffin oil. Although, the selection of high and low performers was biased for olfactory sensitivity, the differential conditioning procedure demonstrates that learning and memory are specific. The brains of the high performers present differences in several neurotransmitter systems, components involved in synaptic transmission and in signaling pathways that are important for learning and memory. Further investigations are needed to describe more precisely the differences between the young and old foragers.

Supporting information

S1 Fig. RT-qPCR analysis of the nine-candidate genes for the high and low performer bees. RT-qPCR analysis of expression differences of the 9-selected gene candidates (represented by respective standard IDs): Comparison of the mRNA levels of the selected genes between the high and low performer bees. Each bar represents the relative mRNA levels +/- SE in the low (green) and high (red) performers, normalized to the levels of the low performers. The differences in expression levels measured by microarray are also given (yellow bars).
(DOCX)

S1 Table. List of primer sequences for the selected transcripts used for RT-qPCR analysis.
(DOCX)

S2 Table. Results of the repeated measures ANOVA for the four sets of retention tests of the pooled population. Fisher statistic values (Stimulus × Trial interaction effect) and *p*-values of the Tukey HSD posthoc test (after Bonferroni correction of *p*-value) are given for the four sets of retention test, performed during the two phases of sequential learning. All *p*-values, except one, represented in bold and with asterisk, are significant. Note that the *p*-value is non-significant (bold and asterisk) for the highest dilutions of the odor pair, OA and LA only during the 4th retention test.
(DOCX)

S3 Table. Results of the repeated measures ANOVA for the two phases of conditioning and four sets of retention tests for the high and low performer bees. Fisher statistic values (Stimulus × Trial interaction effect and Stimulus main effect are shown respectively for the two sets of differential conditioning and four sets of retention tests) and *p*-values of the Tukey HSD posthoc test are given for the high and low performer bees. Both the high and low performer bees showed significant Stimulus × Trial and Stimulus effects however, no significant difference is found between the conditioned responses to the CS+ and CS- odors during the first differential conditioning for the low performers. Bonferroni-corrected *p*-values (Differential conditioning: $p < 0.008$, Retention test: $p < 0.016$) are used for comparisons in the posthoc test. Significant and non-significant *p*-values (posthoc test) are respectively represented with asterisks and in bold (see Fig 5). For the differential conditioning, only the trials with significant *p*-values (posthoc test) are given.
(DOCX)

S4 Table. Spearman rank order correlation coefficients between the seven variables. Correlation table for the seven variables quantified from the pooled data of bees. High correlation coefficient values (in bold) are found between the following pairs: Acq-1 and DisCond-1 (ρ = 0.88), Acq-1 and P-score (ρ = 0.85), DisCond-1 and P-score (ρ = 0.85), DisT-1,-2 and P-score (ρ = 0.7) and Acq-2 and P-score (ρ = 0.69). All correlations are significant (**correlations are significant at the 0.01 level, *correlations are significant at the 0.05 level).
(DOCX)

S5 Table. High performer bees for the analysis of colony effect. Selection of the high performer bees, from the four colonies, from the different months of the experimental year (2010) for the analysis of colony effect. The column 'Bee' is showing the identification number of the bees.
(DOCX)

S6 Table. Behavioral list of candidate genes with changes in expression levels between the high and low performer bees. It shows the sorted behavioral list of 652 candidate genes.
(XLSX)

S7 Table. Analysis of the KAAS output. The analysis of the KAAS output showed 600 enriched terms with diverse metabolic pathways, including classical neurotransmission pathways, olfactory pathways and other signaling pathways.
(XLSX)

S8 Table. DAVID functional annotation chart. The DAVID functional annotation chart showed a list of 76 statistically significant terms from the list of behavioral genes, which

includes microtubule-associated complex, neurogenesis, mitochondrion, etc.
(XLSX)

S9 Table. List of bees selected for the validation by RT-qPCR. The list of selected bees (high and low performers), for the validation by reverse transcription quantitative real-time PCR, from the four colonies and from different months of the experimental year.
(DOCX)

Acknowledgments

We are grateful for the collaboration with Dr. Evren Pamir in the course of analyzing our data. We also thank Mr. Peter Knoll for the help with the bees. We acknowledge the collaboration with Prof. Kaspar Bienefeld, Mr. Fred Zautke, and Dr. Caspar Schoening from the Länderinstitut für Bienenkunde Hohen Neuendorf e.V. for creating the beelines and delivering them to our institute. We would like to thank Dr. Torsten Stein for the revision of the English language.

Author Contributions

Conceptualization: Gérard Leboulle, Randolf Menzel.

Data curation: Neloy Kumar Chakroborty, Gérard Leboulle, Ralf Einspanier, Randolf Menzel.

Formal analysis: Neloy Kumar Chakroborty, Gérard Leboulle, Ralf Einspanier.

Investigation: Neloy Kumar Chakroborty, Gérard Leboulle.

Methodology: Gérard Leboulle, Ralf Einspanier.

Writing – original draft: Neloy Kumar Chakroborty, Gérard Leboulle, Randolf Menzel.

References

1. Menzel R. The honeybee as a model for understanding the basis of cognition. Nature Reviews Neuroscience. 2012; 13(11):758–68. https://doi.org/10.1038/nrn3357 PMID: 23080415
2. Giurfa M. Learning and cognition in insects. Wiley Interdisciplinary Reviews: Cognitive Science. 2015; 6(4):383–95. https://doi.org/10.1002/wcs.1348 PMID: 26263427
3. Lee JJ, Wedow R, Okbay A, Kong E, Maghzian O, Zacher M, et al. Gene discovery and polygenic prediction from a genome-wide association study of educational attainment in 1.1 million individuals. Nature genetics. 2018; 50(8):1112–21. https://doi.org/10.1038/s41588-018-0147-3 PMID: 30038396
4. Miller SE, Legan AW, Henshaw MT, Ostevik KL, Samuk K, Uy FM, et al. Evolutionary dynamics of recent selection on cognitive abilities. Proceedings of the National Academy of Sciences. 2020; 117 (6):3045–52. https://doi.org/10.1073/pnas.1918592117 PMID: 31980529
5. Chittka L, Skorupski P. Information processing in miniature brains. The Royal Society; 2011. p. 885–8. https://doi.org/10.1098/rspb.2010.2699 PMID: 21227971
6. Hill WD, Davies G, Van De Lagemaat LN, Christoforou A, Marioni R, Fernandes C, et al. Human cognitive ability is influenced by genetic variation in components of postsynaptic signalling complexes assembled by NMDA receptors and MAGUK proteins. Translational psychiatry. 2014; 4(1):e341-e. https://doi.org/10.1038/tp.2013.114 PMID: 24399044
7. Schradin C. Intraspecific variation in social organization by genetic variation, developmental plasticity, social flexibility or entirely extrinsic factors. Philosophical Transactions of the Royal Society B: Biological Sciences. 2013; 368(1618):20120346. https://doi.org/10.1098/rstb.2012.0346 PMID: 23569294
8. Lucon-Xiccato T, Carere C, Baracchi D. Intraspecific variation in invertebrate cognition: a review. Behavioral Ecology and Sociobiology. 2024; 78(1):1. https://doi.org/10.1007/s00265-023-03413-8
9. Laskowski KL, Chang CC, Sheehy K, Aguiñaga J. Consistent individual behavioral variation: what do we know and where are we going?. Annual Review of Ecology, Evolution, and Systematics. 2022; 53:161–82. https://doi.org/10.1146/annurev-ecolsys-102220-011451

10. Guenther A, Brust V. Individual consistency in multiple cognitive performance: behavioural versus cognitive syndromes. Animal Behaviour. 2017; 130:119–31. https://doi.org/10.1016/j.anbehav.2017.06.011

11. Dougherty LR, Guillette LM. Linking personality and cognition: a meta-analysis. Philosophical Transactions of the Royal Society B: Biological Sciences. 2018; 373(1756):20170282. https://doi.org/10.1098/rstb.2017.0282 PMID: 30104427

12. Cauchoix M, Chow P, Van Horik J, Atance C, Barbeau E, Barragan-Jason G, et al. The repeatability of cognitive performance: a meta-analysis. Philosophical Transactions of the Royal Society B: Biological Sciences. 2018; 373(1756):20170281. https://doi.org/10.1098/rstb.2017.0281 PMID: 30104426

13. Ashton BJ, Thornton A, Cauchoix M, Ridley AR. Long-term repeatability of cognitive performance. Royal Society Open Science. 2022; 9(5):220069. https://doi.org/10.1098/rsos.220069 PMID: 35620015

14. Lucon-Xiccato T, Bisazza A. Individual differences in cognition among teleost fishes. Behavioural processes. 2017; 141:184–95. https://doi.org/10.1016/j.beproc.2017.01.015 PMID: 28126389

15. Menzel R, Giurfa M. Cognitive architecture of a mini-brain: the honeybee. Trends in cognitive sciences. 2001; 5(2):62–71. https://doi.org/10.1016/s1364-6613(00)01601-6 PMID: 11166636

16. Perry CJ, Barron AB, Chittka L. The frontiers of insect cognition. Current Opinion in Behavioral Sciences. 2017; 16:111–8.19. https://doi.org/10.1016/j.cobeha.2017.05.011

17. Richter JN, Hochner B, Kuba MJ. Pull or push? Octopuses solve a puzzle problem. PloS one. 2016; 11(3):e0152048. https://doi.org/10.1371/journal.pone.0152048 PMID: 27003439

18. Heisenberg M, Gerber B. Behavioral analysis of learning and memory in Drosophila. 2008. https://doi.org/10.1016/B978-012370509-9.00066-8

19. Jiang H, Hanna E, Gatto CL, Page TL, Bhuva B, Broadie K. A fully automated Drosophila olfactory classical conditioning and testing system for behavioral learning and memory assessment. Journal of neuroscience methods. 2016; 261:62–74. https://doi.org/10.1016/j.jneumeth.2015.11.030 PMID: 26703418

20. Chabaud M-A, Preat T, Kaiser L. Behavioral characterization of individual olfactory memory retrieval in Drosophila melanogaster. Frontiers in behavioral neuroscience. 2010; 4:192. https://doi.org/10.3389/fnbeh.2010.00192 PMID: 21258642

21. Quinn WG, Harris WA, Benzer S. Conditioned behavior in Drosophila melanogaster. Proceedings of the National Academy of Sciences. 1974; 71(3):708–12. https://doi.org/10.1073/pnas.71.3.708 PMID: 4207071

22. Brandes C. Estimation of heritability of learning behaviour in honeybees (Apis mellifera capensis). Behavior genetics. 1988; 18:119–32. https://doi.org/10.1007/BF01067081 PMID: 3365194

23. Tait C, Mattise-Lorenzen A, Lark A, Naug D. Interindividual variation in learning ability in honeybees. Behavioural processes. 2019; 167:103918. https://doi.org/10.1016/j.beproc.2019.103918 PMID: 31351114

24. Galsworthy MJ, Paya-Cano JL, Liu L, Monleón S, Gregoryan G, Fernandes C, et al. Assessing reliability, heritability and general cognitive ability in a battery of cognitive tasks for laboratory mice. Behavior genetics. 2005; 35:675–92. https://doi.org/10.1007/s10519-005-3423–9 PMID: 16184494

25. Deaner RO, Van Schaik CP, Johnson V. Do some taxa have better domain-general cognition than others? A meta-analysis of nonhuman primate studies. Evolutionary Psychology. 2006; 4 (1):147470490600400114. https://doi.org/10.1177/147470490600400114

26. Reader SM, Hager Y, Laland KN. The evolution of primate general and cultural intelligence. Philosophical Transactions of the Royal Society B: Biological Sciences. 2011; 366(1567):1017–27. https://doi.org/10.1098/rstb.2010.0342 PMID: 21357224

27. Bitterman ME, Menzel R, Fietz A, Schäfer S. Classical conditioning of proboscis extension in honeybees (Apis mellifera). Journal of Comparative Psychology. 1983; 97:107–19. https://doi.org/10.1037//0735-7036.97.2.107 PMID: 6872507

28. Menzel R, Giurfa M. Dimensions of Cognition in an Insect, the Honeybee. Behavioral and Cognitive Neuroscience Reviews. 2006; 5:24–40. https://doi.org/10.1177/1534582306289522 PMID: 16816091

29. Gallistel CR, Fairhurst S, Balsam P. The learning curve: implications of a quantitative analysis. Proceedings of the National Academy of Sciences. 2004; 101(36):13124–31. https://doi.org/10.1073/pnas.0404965101 PMID: 15331782

30. Pamir E, Szyszka P, Scheiner R, Nawrot MP. Rapid learning dynamics in individual honeybees during classical conditioning. Frontiers in Behavioral Neuroscience. 2014; 8:313. https://doi.org/10.3389/fnbeh.2014.00313 PubMed PMID: PMC4164006. https://doi.org/10.1101/lm.2232711 PMID: 25309366

31. Pamir E, Chakroborty NK, Stollhoff N, Gehring KB, Antemann V, Morgenstern L, et al. Average group behavior does not represent individual behavior in classical conditioning of the honeybee. Learn Mem. 2011; 18(11):733–41. https://doi.org/10.1101/lm.2232711 PMID: 22042602

32. Bloch G, Toma DP, Robinson GE. Behavioral rhythmicity, age, division of labor and period expression in the honey bee brain. Journal of Biological Rhythms. 2001; 16(5):444–56. https://doi.org/10.1177/074873001129002123 PMID: 11669418

33. Shapira M, Thompson CK, Soreq H, Robinson GE. Changes in neuronal acetylcholinesterase gene expression and division of labor in honey bee colonies. Journal of Molecular Neuroscience. 2001; 17:1–12. https://doi.org/10.1385/JMN:17:1:1 PMID: 11665858

34. Zayed A, Naeger NL, Rodriguez-Zas SL, Robinson GE. Common and novel transcriptional routes to behavioral maturation in worker and male honey bees. Genes, Brain and Behavior. 2012; 11(3):253–61. https://doi.org/10.1111/j.1601-183X.2011.00750.x PMID: 22050787

35. Naeger NL, Van Nest BN, Johnson JN, Boyd SD, Southey BR, Rodriguez-Zas SL, Moore D, Robinson GE. Neurogenomic signatures of spatiotemporal memories in time-trained forager honey bees. Journal of Experimental Biology. 2011; 214(6):979–87. https://doi.org/10.1242/jeb.053421 PMID: 21346126

36. Naeger NL, Robinson GE. Transcriptomic analysis of instinctive and learned reward-related behaviors in honey bees. Journal of Experimental Biology. 2016; 219(22):3554–61. https://doi.org/10.1242/jeb.144311 PMID: 27852762

37. Manfredini F, Wurm Y, Sumner S, Leadbeater E. Transcriptomic responses to location learning by honeybee dancers are partly mirrored in the brains of dance-followers. Proceedings of the Royal Society B. 2023; 290(2013):20232274. https://doi.org/10.1098/rspb.2023.2274 PMID: 38113935

38. Jones BM, Rao VD, Gernat T, Jagla T, Cash-Ahmed AC, Rubin BE, et al. Individual differences in honey bee behavior enabled by plasticity in brain gene regulatory networks. Elife. 2020; 9:e62850. https://doi.org/10.7554/eLife.62850 PMID: 33350385

39. Robinson GE, Page RE Jr. Genetic determination of guarding and undertaking in honey-bee colonies. Nature. 1988; 333(6171):356–8. https://doi.org/10.1038/333356a0

40. Lutz CC, Rodriguez-Zas SL, Fahrbach SE, Robinson GE. Transcriptional response to foraging experience in the honey bee mushroom bodies. Dev Neurobiol. 2012; 72(2):153–66. https://doi.org/10.1002/dneu.20929 PMID: 21634017

41. Whitfield CW, Cziko AM, Robinson GE. Gene expression profiles in the brain predict behavior in individual honey bees. Science. 2003; 302(5643):296–9. https://doi.org/10.1126/science.1086807 PMID: 14551438

42. Southey BR, Zhu P, Carr-Markell MK, Liang ZS, Zayed A, Li R, et al. Characterization of genomic variants associated with scout and recruit behavioral castes in honey bees using whole-genome sequencing. PloS one. 2016; 11(1):e0146430. https://doi.org/10.1371/journal.pone.0146430 PMID: 26784945

43. Gempe T, Stach S, Bienefeld K, Otte M, Beye M. Behavioral and molecular studies of quantitative differences in hygienic behavior in honeybees. BMC Research Notes. 2016; 9(1):1–8. https://doi.org/10.1186/s13104-016-2269-y PMID: 27769283

44. El Hassani AK, Schuster S, Dyck Y, Demares F, Leboulle G, Armengaud C. Identification, localization and function of glutamate-gated chloride channel receptors in the honeybee brain. Eur J Neurosci. 2012; 36(4):2409–20. https://doi.org/10.1111/j.1460-9568.2012.08144.x PMID: 22632568

45. Fiala A, Müller U, Menzel R. Reversible downregulation of PKA during olfatory learning using antisense technique impairs long-term memory formation in the honeybee, *Apis mellifera*. Journal of Neuroscience. 1999; 19(22):10125–34. https://doi.org/10.1523/JNEUROSCI.19-22-10125.1999 PMID: 10559420

46. Müßig L, Richlitzki A, Rößler R, Eisenhardt D, Menzel R, Leboulle G. Acute disruption of the NMDA receptor subunit NR1 in the honeybee brain selectively impairs memory formation. Journal of Neuroscience. 2010; 30(23):7817–25. https://doi.org/10.1523/JNEUROSCI.5543-09.2010 PMID: 20534830

47. Toth AL, Varala K, Henshaw MT, Rodriguez-Zas SL, Hudson ME, Robinson GE. Brain transcriptomic analysis in paper wasps identifies genes associated with behaviour across social insect lineages. Proceedings of the Royal Society B: Biological Sciences. 2010; 277(1691):2139–48. https://doi.org/10.1098/rspb.2010.0090 PMID: 20236980

48. Helmkampf M, Mikheyev AS, Kang Y, Fewell J, Gadau J. Gene expression and variation in social aggression by queens of the harvester ant Pogonomyrmex californicus. Molecular ecology. 2016; 25(15):3716–30. https://doi.org/10.1111/mec.13700 PMID: 27178446

49. Menzel R. Memory dynamics in the honeybee. J Comp Physiol [A]. 1999; 185:323–40. https://doi.org/10.1007/s003590050392

50. Dubnau J, Grady L, Kitamoto T, Tully T. Disruption of neurotransmission in Drosophila mushroom body blocks retrieval but not acquisition of memory. Nature. 2001; 411(6836):476–80. https://doi.org/10.1038/35078077 PMID: 11373680

51. Felsenberg J, Jacob PF, Walker T, Barnstedt O, Edmondson-Stait AJ, Pleijzier MW, et al. Integration of parallel opposing memories underlies memory extinction. Cell. 2018; 175(3):709–22. e15. https://doi.org/10.1016/j.cell.2018.08.021 PMID: 30245010

52. Haenicke J, Yamagata N, Zwaka H, Nawrot M, Menzel R. Neural Correlates of Odor Learning in the Presynaptic Microglomerular Circuitry in the Honeybee Mushroom Body Calyx. eNeuro. 2018; 5(3): ENEURO. 0128–18.2018. https://doi.org/10.1523/ENEURO.0128-18.2018 PMID: 29938214

53. Bienefeld K, Reinsch N, Thakur R, editors. Selection for uncapping of Varroa infested brood cells in the honeybee (Apis mellifera). Proc 37th Intern Apimondia Congress, Durban Bucharest: Apimondia Publishing House; 2001. ISBN: 0-620-27768-8.

54. Chandra SB, Hosler JS, Smith BH. Heritable variation for latent inhibition and its correlation with reversal learning in honeybees (Apis mellifera). Journal of Comparative Psychology. 2000; 114(1):86. https://doi.org/10.1037/0735-7036.114.1.86 PMID: 10739314

55. Ferguson HJ, Cobey S, Smith BH. Sensitivity to a change in reward is heritable in the honeybee, Apis mellifera. Animal Behaviour. 2001; 61:527–34. https://doi.org/10.1006/anbe.2000.1635

56. Laloi D, Pham-Delegue M-H. Patriline-level variability in olfactory learning in the honey bee. Apidologie. 2010; 41(4):436–42. https://doi.org/10.1051/apido/2009080

57. Matsumoto Y, Menzel R, Sandoz JC, Giurfa M. Revisiting olfactory classical conditioning of the proboscis extension response in honey bees: a step toward standardized procedures. J Neurosci Methods. 2012; 211(1):159–67. https://doi.org/10.1016/j.jneumeth.2012.08.018 PMID: 22960052

58. Stollhoff N, Menzel R, Eisenhardt D. Spontaneous recovery from extinction depends on the reconsolidation of the acquisition memory in an appetitive learning paradigm in the honeybee (Apis mellifera). J Neurosci. 2005; 25(18):4485–92. https://doi.org/10.1523/JNEUROSCI.0117-05.2005 PMID: 15872095

59. Gramacho KP, Spivak M. Differences in olfactory sensitivity and behavioral responses among honey bees bred for hygienic behavior. Behavioral Ecology and Sociobiology. 2003; 54(5):472–9. https://doi.org/10.1007/s00265-003-0643-y

60. Masterman R, Ross R, Mesce K, Spivak M. Olfactory and behavioral response thresholds to odors of diseased brood differ between hygienic and non-hygienic honey bees (Apis mellifera L.). Journal of Comparative Physiology A. 2001; 187(6):441–52. https://doi.org/10.1007/s003590100216 PMID: 11548991

61. Masterman R, Smith B, Spivak M. Brood odor discrimination abilities in hygienic honey bees (Apis mellifera L.) using proboscis extension reflex conditioning. Journal of Insect Behavior. 2000; 13(1):87–101. https://doi.org/10.1023/A:1007767626594

62. Swanson JA, Torto B, Kells SA, Mesce KA, Tumlinson JH, Spivak M. Odorants that induce hygienic behavior in honeybees: identification of volatile compounds in chalkbrood-infected honeybee larvae. Journal of chemical ecology. 2009; 35:1108–16. https://doi.org/10.1007/s10886-009-9683-8 PMID: 19816752

63. Breed MD, Diaz PH, Lucero KD. Olfactory information processing in honeybee, Apis mellifera, nestmate recognition. Animal behaviour. 2004; 68(4):921–8. https://doi.org/10.1016/j.anbehav.2003.10.033

64. Chakroborty NK, Bienefeld K, Menzel R. Odor learning and odor discrimination of bees selected for enhanced hygienic behavior. Apidologie. 2015; 46:499–514. https://doi.org/10.1007/s13592-014-0342-x

65. Lunney GH. Using analysis of variance with a dichotomous dependent variable: An empirical study 1. Journal of educational measurement. 1970; 7(4):263–9. https://doi.org/10.1111/j.1745-3984.1970.tb00727.x

66. Mota T, Giurfa M. Multiple reversal olfactory learning in honeybees. Frontiers in behavioral neuroscience. 2010; 4:48. https://doi.org/10.3389/fnbeh.2010.00048 PMID: 20700501

67. Rath L, Giovanni GC, Szyszka P. Multiple memory traces after associative learning in the honey bee antennal lobe. Eur J Neurosci. 2011; 34(2):352–60. https://doi.org/10.1111/j.1460-9568.2011.07753.x PMID: 21692886

68. Whitfield CW, Ben-Shahar Y, Brillet C, Leoncini I, Crauser D, LeConte Y, et al. Genomic dissection of behavioral maturation in the honey bee. Proceedings of the National Academy of Sciences. 2006; 103 (44):16068–75. https://doi.org/10.1073/pnas.0606909103 PMID: 17065327

69. Consortium BGS, authors) DEaGLaat. Insights into social insects from the genome of the honeybee, Apis mellifera. Nature. 2006; 443(7114):931–49. https://doi.org/10.1038/nature05260

70. Ritchie ME, Silver J, Oshlack A, Holmes M, Diyagama D, Holloway A, et al. A comparison of background correction methods for two-colour microarrays. Bioinformatics. 2007; 23(20):2700–7. https://doi.org/10.1093/bioinformatics/btm412 PMID: 17720982

71. Smyth GK, Speed T. Normalization of cDNA microarray data. Methods. 2003; 31(4):265–73. https://doi.org/10.1016/s1046-2023(03)00155-5 PMID: 14597310

72. Dennis G, Sherman BT, Hosack DA, Yang J, Gao W, Lane HC, et al. DAVID: database for annotation, visualization, and integrated discovery. Genome biology. 2003; 4(9):1–11. https://doi.org/10.1186/gb-2003-4-9-r60 PMID: 12734009

73. Huang DW, Sherman BT, Tan Q, Collins JR, Alvord WG, Roayaei J, et al. The DAVID Gene Functional Classification Tool: a novel biological module-centric algorithm to functionally analyze large gene lists. Genome biology. 2007; 8(9):1–16. https://doi.org/10.1186/gb-2007-8-9-r183 PMID: 17784955

74. Genath A, Sharbati S, Buer B, Nauen R, Einspanier R. Comparative transcriptomics indicates endogenous differences in detoxification capacity after formic acid treatment between honey bees and varroa mites. Scientific reports. 2020; 10(1):21943. https://doi.org/10.1038/s41598-020-79057-9 PMID: 33318550

75. Sih A, Del Giudice M. Linking behavioural syndromes and cognition: a behavioural ecology perspective. Philosophical Transactions of the Royal Society B: Biological Sciences. 2012; 367(1603):2762–72. https://doi.org/10.1098/rstb.2012.0216 PMID: 22927575

76. Sih A, Bell A, Johnson JC. Behavioral syndromes: an ecological and evolutionary overview. Trends in ecology & evolution. 2004; 19(7):372–8. https://doi.org/10.1016/j.tree.2004.04.009 PMID: 16701288

77. Biro PA, Stamps JA. Are animal personality traits linked to life-history productivity? Trends in ecology & evolution. 2008; 23(7):361–8. https://doi.org/10.1016/j.tree.2008.04.003 PMID: 18501468

78. Maák I, Roelandt G, d'Ettorre P. A small number of workers with specific personality traits perform tool use in ants. Elife. 2020; 9:e61298. https://doi.org/10.7554/eLife.61298 PMID: 33295872

79. Réale D, Reader SM, Sol D, McDougall PT, Dingemanse NJ. Integrating animal temperament within ecology and evolution. Biological reviews. 2007; 82(2):291–318. https://doi.org/10.1111/j.1469-185X.2007.00010.x PMID: 17437562

80. Réale D, Garant D, Humphries MM, Bergeron P, Careau V, Montiglio P-O. Personality and the emergence of the pace-of-life syndrome concept at the population level. Philosophical Transactions of the Royal Society B: Biological Sciences. 2010; 365(1560):4051–63. https://doi.org/10.1098/rstb.2010.0208 PMID: 21078657

81. Scheiner R, Erber J, Page RE, Jr., Tactile learning and the individual evaluation of the reward in honey bees (Apis mellifera L.). J Comp Physiol [A]. 1999; 185:1–10. https://doi.org/10.1007/s003590050360 PMID: 10450609

82. Scheiner R, Reim T, Søvik E, Entler BV, Barron AB, Thamm M. Learning, gustatory responsiveness and tyramine differences across nurse and forager honeybees. Journal of Experimental Biology. 2017; 220(8):1443–50. https://doi.org/10.1242/jeb.152496 PMID: 28167800

83. Pankiw T, Page RE Jr. Response thresholds to sucrose predict foraging division of labor in honeybees. Behavioral ecology and sociobiology. 2000; 47:265–7. https://doi.org/10.1007/s002650050664

84. Finke V, Baracchi D, Giurfa M, Scheiner R, Avarguès-Weber A. Evidence of cognitive specialization in an insect: proficiency is maintained across elemental and higher-order visual learning but not between sensory modalities in honey bees. Journal of Experimental Biology. 2021; 224(24):jeb242470. https://doi.org/10.1242/jeb.242470 PMID: 34664669

85. Finke V, Scheiner R, Giurfa M, Avarguès-Weber A. Individual consistency in the learning abilities of honey bees: cognitive specialization within sensory and reinforcement modalities. Animal Cognition. 2023; 26(3):909–28. https://doi.org/10.1007/s10071-022-01741–2 PMID: 36609813

86. Fernandez PC, Locatelli FF, Person-Rennell N, Deleo G, Smith BH. Associative conditioning tunes transient dynamics of early olfactory processing. J Neurosci. 2009; 29(33):10191–202. https://doi.org/10.1523/JNEUROSCI.1874-09.2009 PMID: 19692594

87. Smith BH, Huerta R, Bazhenov M, Sinakevitch I. Distributed plasticity for olfactory learning and memory in the honey bee brain. Honeybee Neurobiology and Behavior: A Tribute to Randolf Menzel. 2012: 393–408. https://doi.org/10.1007/978-94-007-2099-2

88. Guerrieri F, Schubert M, Sandoz J-C, Giurfa M. Perceptual and Neural Olfactory Similarity in Honeybees. PLoS Biology. 2005; 3(4):60–74. https://doi.org/10.1371/journal.pbio.0030060 PMID: 15736975

89. Chandra S, Smith BH. An Analysis of synthetic processing of odor mixtures in the honeybee. Journal of Experimental Biology. 1998; 201:3113–21. https://doi.org/10.1242/jeb.201.22.3113 PMID: 9787131

90. Hayat SA, Luben R, Moore S, Dalzell N, Bhaniani A, Anuj S, et al. Cognitive function in a general population of men and women: a cross sectional study in the European Investigation of Cancer–Norfolk

cohort (EPIC-Norfolk). BMC geriatrics. 2014; 14:1–16. https://doi.org/10.1186/1471-2318-14-142 PMID: 25527303

91. Drezner-Levy T, Smith BH, Shafir S. The effect of foraging specialization on various learning tasks in the honey bee (Apis mellifera). Behavioral Ecology and Sociobiology. 2009; 64:135–48. https://doi.org/10.1007/s00265-009-0829-z

92. Vergoz V, Schreurs HA, Mercer AR. Queen pheromone blocks aversive learning in young worker bees. Science. 2007; 317(5836):384–6. https://doi.org/10.1126/science.1142448 PMID: 17641204

93. Ten Cate C, Rowe C. Biases in signal evolution: learning makes a difference. Trends in ecology & evolution. 2007; 22(7):380–7. https://doi.org/10.1016/j.tree.2007.03.006 PMID: 17379354

94. Niggebrugge C, Leboulle G, Menzel R, Komischke B, de Ibarra NH. Fast learning but coarse discrimination of colours in restrained honeybees. J Exp Biol. 2009; 212(Pt 9):1344–50. https://doi.org/10.1242/jeb.021881 PMID: 19376955

95. Evans LJ, Smith KE, Raine NE. Fast learning in free-foraging bumble bees is negatively correlated with lifetime resource collection. Scientific Reports. 2017; 7(1):496. https://doi.org/10.1038/s41598-017-00389-0 PMID: 28356567

96. Brandes C, Frisch B, Menzel R. Time-course of memory formation differs in honey bee lines selected for good and poor learning. Anim Behav. 1988; 36:981–5. https://doi.org/10.1016/S0003-3472(88)80056-3

97. Deanhardt B, Duan Q, Du C, Soeder C, Morlote A, Garg D, et al. Social experience and pheromone receptor activity reprogram gene expression in sensory neurons. G3: Genes, Genomes, Genetics. 2023; 13(6):jkad072. https://doi.org/10.1093/g3journal/jkad072 PMID: 36972331

98. Veatch OJ, Keenan BT, Gehrman PR, Malow BA, Pack AI. Pleiotropic genetic effects influencing sleep and neurological disorders. The Lancet Neurology. 2017; 16(2):158–70. https://doi.org/10.1016/S1474-4422(16)30339-8 PMID: 28102151

99. Kamboh MI. Genomics and functional genomics of Alzheimer's disease. Neurotherapeutics. 2022; 19(1):152–72. https://doi.org/10.1007/s13311-021-01152-0 PMID: 34935119

100. Kucharski R, Maleszka R, Hayward D, Ball E. A royal jelly protein is expressed in a subset of Kenyon cells in the mushroom bodies of the honey bee brain. Naturwissenschaften. 1998; 85(7):343–6. https://doi.org/10.1007/s001140050512 PMID: 9722965

101. Ueno T, Nakaoka T, Takeuchi H, Kubo T. Differential gene expression in the hypopharyngeal glands of worker honeybees (Apis mellifera L.) associated with an age-dependent role change. Zoological science. 2009; 26(8):557–63. https://doi.org/10.2108/zsj.26.557 PMID: 19719408

102. Buttstedt A, Moritz RF, Erler S. More than royal food-Major royal jelly protein genes in sexuals and workers of the honeybee Apis mellifera. Frontiers in zoology. 2013; 10:1–10. https://doi.org/10.1186/1742-9994-10-72 PMID: 24279675

103. Ohashi K, Natori S, Kubo T. Expression of amylase and glucose oxidase in the hypopharyngeal gland with an age-dependent role change of the worker honeybee (Apis mellifera L.). European Journal of Biochemistry. 1999; 265(1):127–33. https://doi.org/10.1046/j.1432-1327.1999.00696.x PMID: 10491166

104. Vannette RL, Mohamed A, Johnson BR. Forager bees (Apis mellifera) highly express immune and detoxification genes in tissues associated with nectar processing. Scientific reports. 2015; 5(1):16224. https://doi.org/10.1038/srep16224 PMID: 26549293

105. Knecht D, Kaatz H. Patterns of larval food production by hypopharyngeal glands in adult worker honey bees. Apidologie. 1990; 21(5):457–68. https://doi.org/10.1051/apido:19900507

106. Peters L, Zhu-Salzman K, Pankiw T. Effect of primer pheromones and pollen diet on the food producing glands of worker honey bees (Apis mellifera L.). Journal of insect physiology. 2010; 56(2):132–7. https://doi.org/10.1016/j.jinsphys.2009.09.014 PMID: 19799907

107. Kamakura M. Royalactin induces queen differentiation in honeybees. Nature. 2011; 473(7348):478–83. https://doi.org/10.1038/nature10093 PMID: 21516106

108. Ahmad S, Khan SA, Khan KA, Li J. Novel insight into the development and function of hypopharyngeal glands in honey bees. Frontiers in Physiology. 2021; 11:615830. https://doi.org/10.3389/fphys.2020.615830 PMID: 33551843

109. Ben-Shahar Y, Robichon A, Sokolowski M, Robinson G. Influence of gene action across different time scales on behavior. Science. 2002; 296(5568):741–4. https://doi.org/10.1126/science.1069911 PMID: 11976457

110. Ben-Shahar Y, Dudek NL, Robinson GE. Phenotypic deconstruction reveals involvement of manganese transporter malvolio in honey bee division of labor. Journal of experimental biology. 2004; 207(19):3281–8. https://doi.org/10.1242/jeb.01151 PMID: 15326204

111. Orgad S, Nelson H, Segal D, Nelson N. Metal ions suppress the abnormal taste behavior of the Drosophila mutant malvolio. Journal of Experimental Biology. 1998; 201(1):115–20. https://doi.org/10.1242/jeb.201.1.115 PMID: 9390942

112. Alaux C, Kemper N, Kretzschmar A, Le Conte Y. Brain, physiological and behavioral modulation induced by immune stimulation in honeybees (Apis mellifera): A potential mediator of social immunity? Brain, behavior, and immunity. 2012; 26(7):1057–60. https://doi.org/10.1016/j.bbi.2012.04.004 PMID: 22542419

113. Ray S, Ferneyhough B. Behavioral development and olfactory learning in the honeybee (Apis mellifera). Developmental Psychobiology: The Journal of the International Society for Developmental Psychobiology. 1999; 34(1):21–7. https://doi.org/10.1002/(SICI)1098-2302(199901)34:1<21::AID-DEV4>3.0.CO;2-K PMID: 9919430

114. Corona M, Branchiccela B, Alburaki M, Palmer-Young EC, Madella S, Chen Y, et al. Decoupling the effects of nutrition, age, and behavioral caste on honey bee physiology, immunity, and colony health. Frontiers in Physiology. 2023; 14:1149840. https://doi.org/10.3389/fphys.2023.1149840 PMID: 36994419

115. Maleszka J, Barron AB, Helliwell PG, Maleszka R. Effect of age, behaviour and social environment on honey bee brain plasticity. https://doi.org/10.1007/s00359-009-0449-0J Comp Physiol A Neuroethol Sens Neural Behav Physiol. 2009; 195(8):733–40.

116. Scheiner R, Baumann A, Blenau W. Aminergic control and modulation of honeybee behaviour. Current neuropharmacology. 2006; 4(4):259–76. https://doi.org/10.2174/157015906778520791 PMID: 18654639

117. Thamm M, Scheiner R. PKG in honey bees: spatial expression, Amfor gene expression, sucrose responsiveness, and division of labor. Journal of Comparative Neurology. 2014; 522(8):1786–99. https://doi.org/10.1002/cne.23500 PMID: 24214291
